# A taxonomic signature of obesity in a large study of American adults

**DOI:** 10.1038/s41598-018-28126-1

**Published:** 2018-06-27

**Authors:** Brandilyn A. Peters, Jean A. Shapiro, Timothy R. Church, George Miller, Chau Trinh-Shevrin, Elizabeth Yuen, Charles Friedlander, Richard B. Hayes, Jiyoung Ahn

**Affiliations:** 1 Department of Population Health, New York University School of Medicine, New York, NY USA; 2 Division of Cancer Prevention and Control, Centers for Disease Control and Prevention, Atlanta, GA USA; 3 Division of Environmental Health Sciences, School of Public Health, University of Minnesota, Minneapolis, MN USA; 4 Department of Surgery, New York University School of Medicine, New York, NY USA; 5 Department of Cell Biology, New York University School of Medicine, New York, NY USA; 6 NYU Perlmutter Cancer Center, New York University School of Medicine, New York, NY USA; 7 Kips Bay Endoscopy Center, New York, NY USA

## Abstract

Animal models suggest that gut microbiota contribute to obesity; however, a consistent taxonomic signature of obesity has yet to be identified in humans. We examined whether a taxonomic signature of obesity is present across two independent study populations. We assessed gut microbiome from stool for 599 adults, by 16S rRNA gene sequencing. We compared gut microbiome diversity, overall composition, and individual taxon abundance for obese (BMI ≥ 30 kg/m^2^), overweight (25 ≤ BMI < 30), and healthy-weight participants (18.5 ≤ BMI < 25). We found that gut species richness was reduced (p = 0.04), and overall composition altered (p = 0.04), in obese (but not overweight) compared to healthy-weight participants. Obesity was characterized by increased abundance of class Bacilli and its families Streptococcaceae and Lactobacillaceae, and decreased abundance of several groups within class Clostridia, including Christensenellaceae, Clostridiaceae, and Dehalobacteriaceae (q < 0.05). These findings were consistent across two independent study populations. When random forest models were trained on one population and tested on the other as well as a previously published dataset, accuracy of obesity prediction was good (~70%). Our large study identified a strong and consistent taxonomic signature of obesity. Though our study is cross-sectional and causality cannot be determined, identification of microbes associated with obesity can potentially provide targets for obesity prevention and treatment.

## Introduction

The World Health Organization estimates that global obesity prevalence has more than doubled since 1980, classifying >600 million adults as obese in 2014. Obesity increases risk for many diseases, including cancer, atherosclerosis, and diabetes^1–3^. While the fundamental cause of obesity is an imbalance between energy intake and expenditure, other factors may modify susceptibility, such as genetics^4^, epigenetics^5^, and gut microbial composition^6^. Because of the potential to modify bacterial communities, the microbiome is an enticing candidate to target for obesity prevention and treatment. Reaching this goal requires identification of specific taxa and/or microbial functions associated with obesity in humans; once identified, further downstream experimentation can establish whether these taxa and/or functions are causative agents^7^, and, if so, suggest interventions.

Experiments in germ-free mice colonized with gut microbiota from wild-type mice^8^, obese mice^9^, or obese humans^10^, demonstrate that microbiota play a critical role in adiposity in test systems. Moreover, these experiments have demonstrated transmissibility of obese phenotypes via gut microbes. These findings lead to the question of whether gut microbial composition confers susceptibility to obesity in humans. An early report in a small human sample (n = 14)^11^ was consistent with findings in mice that obesity, whether genetic^12^ or diet-induced^13,14^, is associated with an increase in relative abundance of the Firmicutes phylum, and a decrease in relative abundance of the Bacteroidetes phylum. However, more recent studies in humans have not corroborated this pattern^15–21^. Recent meta-analyses of studies with 16S rRNA gene data have not found consistent obesity-related taxonomic signatures across studies^22–24^. Small sample sizes, heterogeneous populations, insufficient confounder control, and different methodologies may contribute to disagreement between studies.

Using data from two independent cross-sectional studies of older American adults (n = 599), we aimed to: (1) examine whether within-person microbial diversity (α-diversity) and between-person differences in overall microbial composition (β-diversity) are associated with obesity, and (2) identify specific taxa and inferred metagenomic functions associated with obesity. The latter aim may provide targets for research on obesity treatment and prevention.

## Results

### Participant characteristics

Descriptive characteristics of healthy-weight, overweight, and obese participants are presented in Table 1. Participants were initially recruited for a colonoscopy-screening study, and approximately half (48%) had asymptomatic colorectal polyps detected at study screening or a previous screening. Participants were predominantly white (94%) and above middle-age (62 ± 7 years old). The overweight and obese groups had higher percentages of men than the healthy-weight group (p < 0.0001), while race and age distributions did not differ significantly across BMI categories. Data on energy intake and exercise were available in the New York University (NYU) study only. Daily energy intake did not differ significantly across BMI categories, although a weak positive correlation was detected between energy intake and continuous BMI (Spearman r = 0.17, p = 0.02). Additionally, overweight and obese participants exercised less frequently than healthy-weight participants (p = 0.01).

**Table 1:** Characteristics of participants in the CDC and NYU studies by BMI^a^.

	Healthy-weight	Overweight	Obese	*p* ^b^
Combined (n = 599)	n = 211	n = 246	n = 142	
Men (%)	37.9	69.5	49.3	<0.0001
Age (y; mean ± SD)	62.7 ± 7.7	62.1 ± 7.0	61.7 ± 6.1	0.32
Race (%)				0.26
White	95.3	93.9	93.7	
Black	1.4	3.3	4.2	
Other	3.3	2.0	0.7	
Missing	0	0.8	1.4	
Colorectal polyps^c^ (%)	42.7	50.8	51.4	0.15
BMI (kg/m^2^; mean ± SD)	22.6 ± 1.7	27.1 ± 1.4	35.0 ± 5.0	<0.0001
CDC (n = 423)	n = 130	n = 173	n = 120	
Men (%)	35.4	68.8	49.2	<0.0001
Age (y; mean ± SD)	62.8 ± 4.7	62.2 ± 5.1	62.4 ± 4.8	0.50
Race (%)				0.68
White	96.9	97.7	96.7	
Black	1.5	0.6	2.5	
Other	1.5	1.7	0.8	
Missing	0	0	0	
Colorectal polyps (%)	34.6	44.5	50.0	0.04
BMI (kg/m^2^; mean ± SD)	22.7 ± 1.6	27.1 ± 1.4	34.9 ± 5.0	<0.0001
NYU (n = 176)	n = 81	n = 73	n = 22	
Men (%)	42.0	71.2	50.0	0.001
Age (y; mean ± SD)	62.4 ± 10.8	61.8 ± 10.2	57.7 ± 9.9	0.18
Race (%)				0.06
White	92.6	84.9	77.3	
Black	1.2	9.6	13.6	
Other	6.2	2.7	0	
Missing	0	2.8	9.1	
Colorectal polyps (%)	55.6	65.8	59.1	0.43
BMI (kg/m^2^; mean ± SD)	22.3 ± 1.8	27.0 ± 1.4	35.5 ± 5.4	<0.0001
Daily energy intake^d,e^ (kcal; mean ± SD)	1,703 ± 755	1,846 ± 723	1,830 ± 719	0.34
Exercise^d^ (%)				0.01
None	7.4	16.4	27.3	
<1 hr/week	7.4	8.2	27.3	
1 hr/week	11.1	8.2	0	
2 hr/week	11.1	13.7	18.2	
3 hr/week	29.6	24.7	4.5	
4+ hr/week	33.3	28.8	18.2	
Missing	0	0	4.5	

^a^Healthy-weight: 18.5 ≤ BMI < 25 kg/m^2^; Overweight: 25 ≤ BMI < 30 kg/m^2^; Obese: BMI ≥ 30 kg/m^2^.

^b^P-value for difference between BMI categories from Kruskal-Wallis test for continuous variables and *X*^2^ test for categorical variables.

^c^Had one or more colorectal polyps currently or previously identified.

^d^Variable only available in NYU study (n = 171 for energy intake, n = 175 for exercise).

^e^Determined from food frequency questionnaire.

### α- and β-diversity in relation to obesity

**Figure 1:**
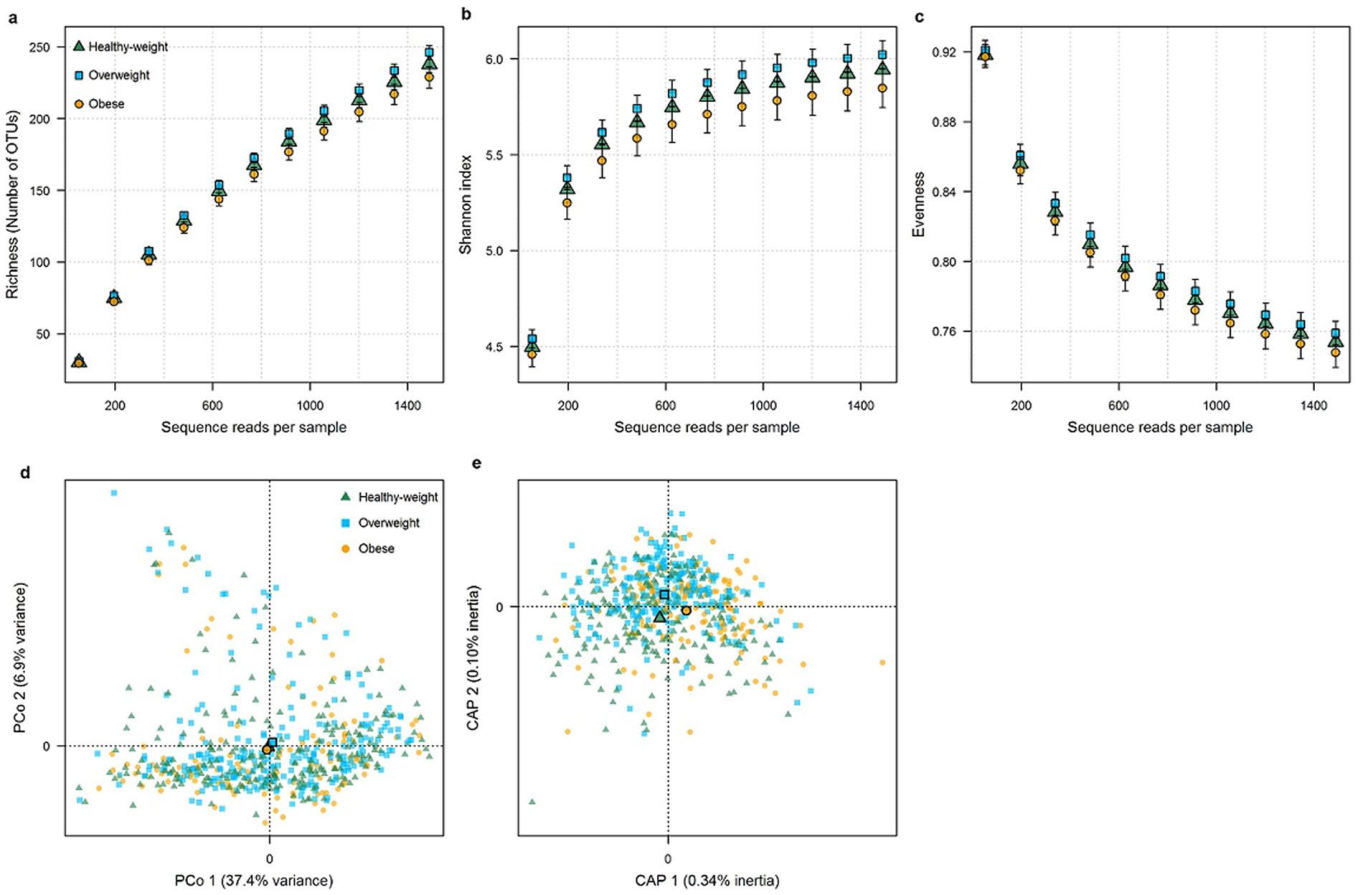
α-diversity and β-diversity in relation to BMI. (**a**–**c**) Richness, Shannon diversity index, and Evenness rarefaction curves in healthy-weight, overweight, and obese participants. Rarefaction curves were estimated by taking the mean of the α-diversity indices averaged for each participant over 100 iterations at each rarefaction sequencing depth. (**d**) Principal coordinate analysis of the weighted UniFrac distances. Shapes outlined in black represent centroids for healthy-weight, overweight, and obese participants. (**e**) Partial constrained analysis of principal coordinates (CAP) based on the weighted UniFrac distance. BMI category was the constraining variable, and sex, age, polyp status, and study were conditioning variables.

Globally, BMI category was associated with richness (i.e. number of OTUs) (p = 0.002) and the Shannon index (p = 0.03), but not with evenness (p = 0.14), at a rarefaction depth of 1,490 sequence reads/sample (Supplemental Table 1). In pairwise comparisons, richness was reduced in obese compared to healthy-weight participants (b = −9.87, p = 0.04, p_Holm_ = 0.08); this pattern was apparent, though not statistically significant, for the Shannon index (b = −0.11, p = 0.11, p_Holm_ = 0.22) and evenness (b = −0.01, p = 0.22, p_Holm_ = 0.44) (Fig. 1a–c; Supplemental Table 1). Overweight participants did not differ significantly from healthy-weight participants for any of these α-diversity indices (Supplemental Table 1). Partial constrained analysis of principal coordinates (CAP) of the weighted UniFrac distance revealed separation of obese from both healthy-weight and overweight participants on the main axis, with overweight separated from healthy-weight participants on the secondary axis (Fig. 1e), although principal coordinate analysis (PCoA) did not reveal clustering by BMI category (Fig. 1d). In permutational multivariate analysis of variance (PERMANOVA) analysis of the weighted UniFrac distance, BMI category was not associated globally with overall microbiome composition (p = 0.14). In pairwise comparisons, overall microbiome composition differed between obese and healthy-weight participants (p = 0.04, p_Holm_ = 0.07), while overweight and healthy-weight participants did not differ significantly (p = 0.64, p_Holm_ = 0.64) (Supplemental Table 1). When further classifying obese participants as class I (30 < BMI ≤ 35 kg/m^2^; n = 90) or class II-III (BMI > 35 kg/m^2^; n = 52), we observed that both classes of obesity tended to differ from healthy-weight participants in richness and overall microbiome composition, though not with statistical significance (Supplemental Fig. 1; Supplemental Table 1).

The relationship of obesity with overall microbiome diversity and composition was consistent in both the Centers for Disease Control and Prevention (CDC) and NYU studies, and in those with and without asymptomatic colorectal polyps (Supplemental Fig. 2a,b; Supplemental Table 2). We observed a significant reduction in richness in obese vs. healthy-weight women (p = 0.03), however this was not observed in men (p = 0.47) (Supplemental Fig. 2a; Supplemental Table 2). In the NYU study, availability of diet (n = 171) and exercise (n = 175) data allowed us to assess whether exercise or intake of total energy, fiber, fat, or protein confounded the association of obesity with microbiome diversity and composition. We observed that adjustment for these variables did not attenuate differences in diversity and composition between obese and healthy-weight participants in the NYU study (Supplemental Table 3).

### Taxa associated with obesity

**Figure 2:**
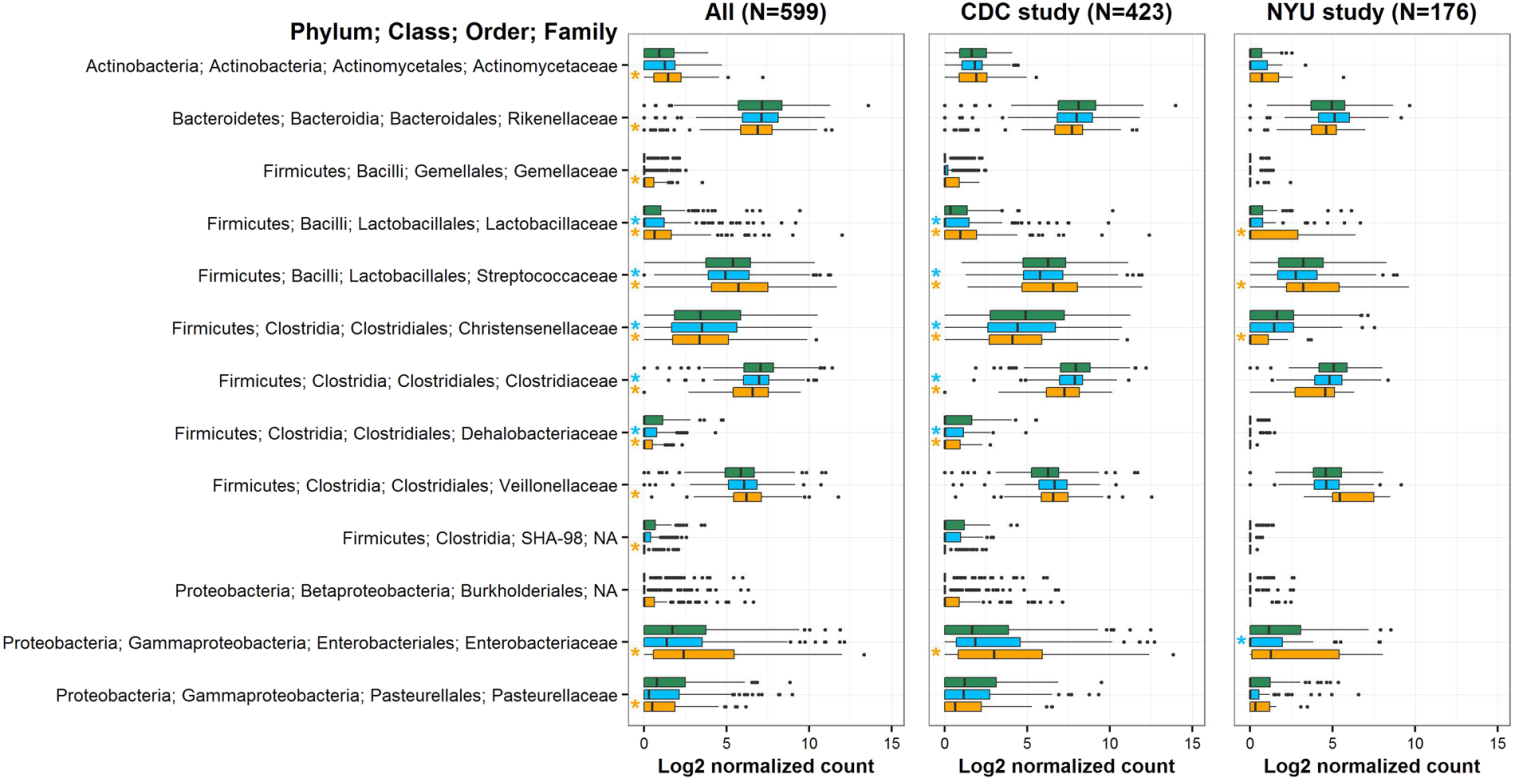
Count boxplots of families that were differentially abundant by obesity. Families associated globally with BMI category in the DESeq2 analysis (LRT q < 0.05) were included in the plot. Green, blue, and orange boxplots represent healthy-weight, overweight, and obese participants, respectively. Counts were normalized for DESeq2 size factors and log2 transformed after adding a pseudocount of 1. Stars to the left-hand side of boxplots indicate significant difference in abundance from healthy-weight (p_Holm_ < 0.05 for pairwise comparison).

We examined differential abundance of taxa by BMI at the phylum through OTU levels (Supplemental Table 4). Contrary to several previous reports, abundances of the two most prevalent phyla, Firmicutes and Bacteroidetes, were not associated with BMI category (p = 0.40 and p = 0.49, respectively). The Firmicutes/Bacteroidetes ratio was also not associated with BMI category (Kruskal-Wallis test p = 0.94). However, several sub-taxa within Firmicutes were associated with obesity. The Bacilli class (fold change [FC] = 2.93) and its Streptococcaceae (FC = 2.42), Lactobacillaceae (FC = 6.23), and Gemellaceae (FC = 2.3) families were elevated in obese compared to healthy-weight participants. Within class Clostridia, the Christensenellaceae (FC = 0.57), Clostridiaceae (FC = 0.58), Dehalobacteriaceae (FC = 0.34), and SHA-98 (FC = 0.49) families were depleted, and the Veillonellaceae family enriched (FC = 1.46), in obese compared to healthy-weight participants. Greater abundances of family Actinomycetaceae of phylum Actinobacteria, and family Enterobacteriaceae of phylum Proteobacteria, were also noted in obese participants, as were decreased abundances of family Rikenellaceae (Bacteroidetes phylum) and Pasteurellaceae (Proteobacteria phylum) (Fig. 2; Supplemental Table 4). Similar to findings in obese participants, overweight participants had increased abundance of Lactobacillaceae and Streptococcaceae, and decreased abundance of Christensenellaceae, Clostridiaceae, and Dehalobacteriaceae, compared to healthy-weight participants (Fig. 2; Supplemental Table 4).

**Figure 3:**
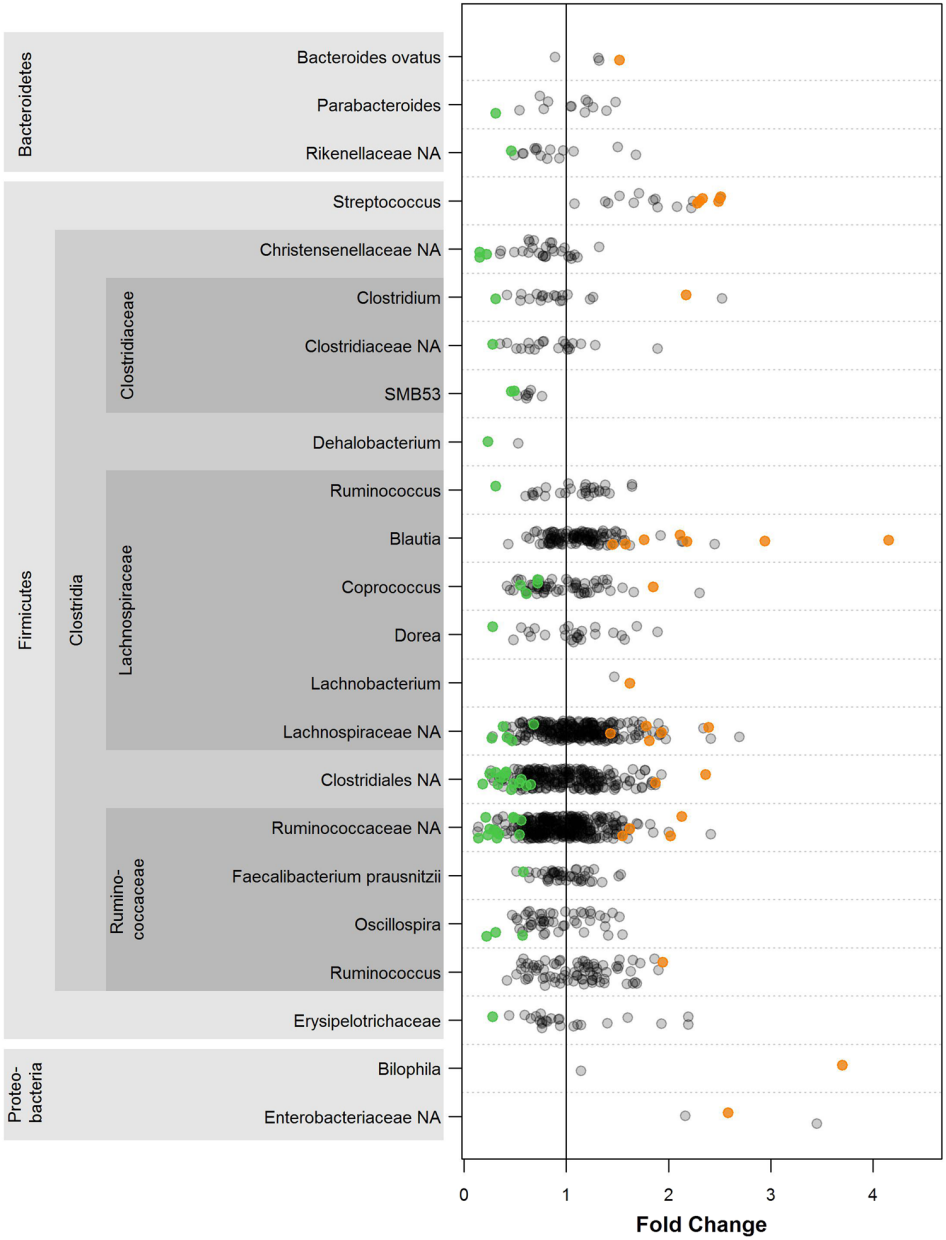
OTUs associated with obesity. OTU fold changes for obese vs. healthy-weight comparison in DESeq2 analysis are plotted. All OTUs within the given taxonomic groups are plotted, and orange and green points represent OTUs significantly (p_Holm_ < 0.05) higher or lower in abundance, respectively, in obese compared to healthy-weight participants. Only taxonomic groups with at least one differentially abundant OTU (p_Holm_ < 0.05) are displayed. “NA” indicates a group that was unclassified at the family, genus, or species level.

At OTU level, 90 OTUs were identified as differentially abundant globally by BMI category at q < 0.05 (Fig. 3; Supplemental Table 4). OTUs in *Streptococcus* and Proteobacteria (Enterobacteriaceae and *Bilophila*) were enriched in obese compared to healthy-weight participants. Within Clostridia, several patterns emerged when comparing obese to healthy-weight participants, including enrichment of *Blautia* OTUs, and depletion of *Coprococcus*, *Oscillospira*, Clostridiaceae, Christensenellaceae, and *Dehalobacterium* OTUs, in the obese. Additionally, many unclassified OTUs within Clostridia (Ruminococcaceae and unclassified families) were depleted in the obese. Fewer OTUs were differentially abundant between overweight and healthy-weight participants, though findings were similar to those in obese participants (Supplemental Table 4).

When stratifying these analyses by sex, we observed some similarities between men and women (Supplemental Table 5). For example, obese men and women both had increased Bacilli, *Streptococcus*, and Gammaproteobacteria, and decreased Christensenellaceae, Clostridiaceae, and Dehalobacteriaceae, than healthy-weight men and women, respectively (though not always reaching p_Holm_ < 0.05).

### Inferred metagenome pathways associated with obesity

**Figure 4:**
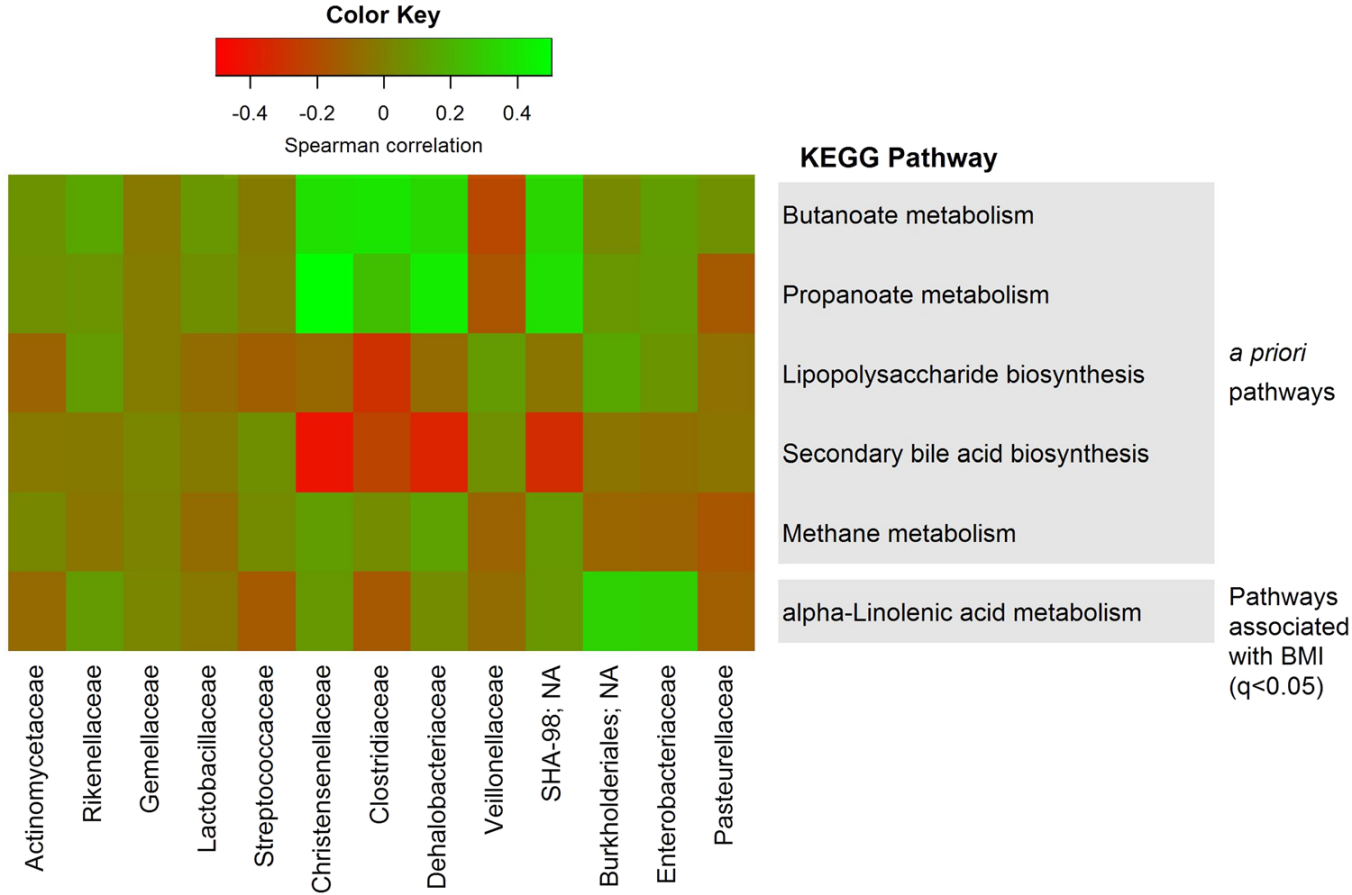
Correlations of bacterial families and inferred metagenomic functions. Family and KEGG pathway counts were DESeq2-normalized. Partial Spearman’s correlation coefficients were estimated for each pairwise comparison of family and KEGG pathway abundance, adjusting for age, sex, study, and polyp status. KEGG pathways included in the heatmap were identified *a priori* or were associated globally with BMI category (LRT q < 0.05); families included in the heatmap were associated globally with BMI category (LRT q < 0.05).

The KEGG pathway “alpha-Linolenic acid (ALA) metabolism” was differentially abundant globally by BMI category (q < 0.05); in pairwise comparisons, this pathway was enriched in obese compared to healthy-weight participants (p < 0.0001, p_Holm_ < 0.0001) (Supplemental Table 6). We also investigated whether several *a priori* pathways, related to hypothesized mechanisms of microbial involvement in obesity (discussed later), were nominally associated with obesity (Supplemental Table 6). “Butanoate (butyrate) metabolism” was marginally depleted (p = 0.06, p_Holm_ = 0.11), while “secondary bile acid biosynthesis” was marginally enriched (p = 0.08, p_Holm_ = 0.17), in obese compared to healthy-weight participants. “Lipopolysaccharide biosynthesis”, “propanoate (propionate) metabolism”, and “methane metabolism” were not associated with obesity. Interestingly, several families depleted in obese compared to healthy-weight participants (Christensenellaceae, Clostridiaceae, Dehalobacteriaceae, and SHA-98) were positively associated with butanoate and propanoate metabolism, and inversely associated with secondary bile acid biosynthesis (Fig. 4). We also explored whether OTUs associated with obesity contributed to abundance of KEGG orthologs for butyrate synthesis genes, butyrate kinase and butyryl-CoA:acetate CoA transferase^25^. While several obesity-depleted OTUs did contribute to butyrate synthesis KEGG orthologs (e.g. OTUs from Christensenellaceae, *Oscillospira*, *SMB53*, Clostridiales, Rikenellaceae), obesity-enriched OTUs also contributed to these orthologs (Supplemental Fig. 3).

### Homogeneity of results across two independent populations

**Figure 5:**
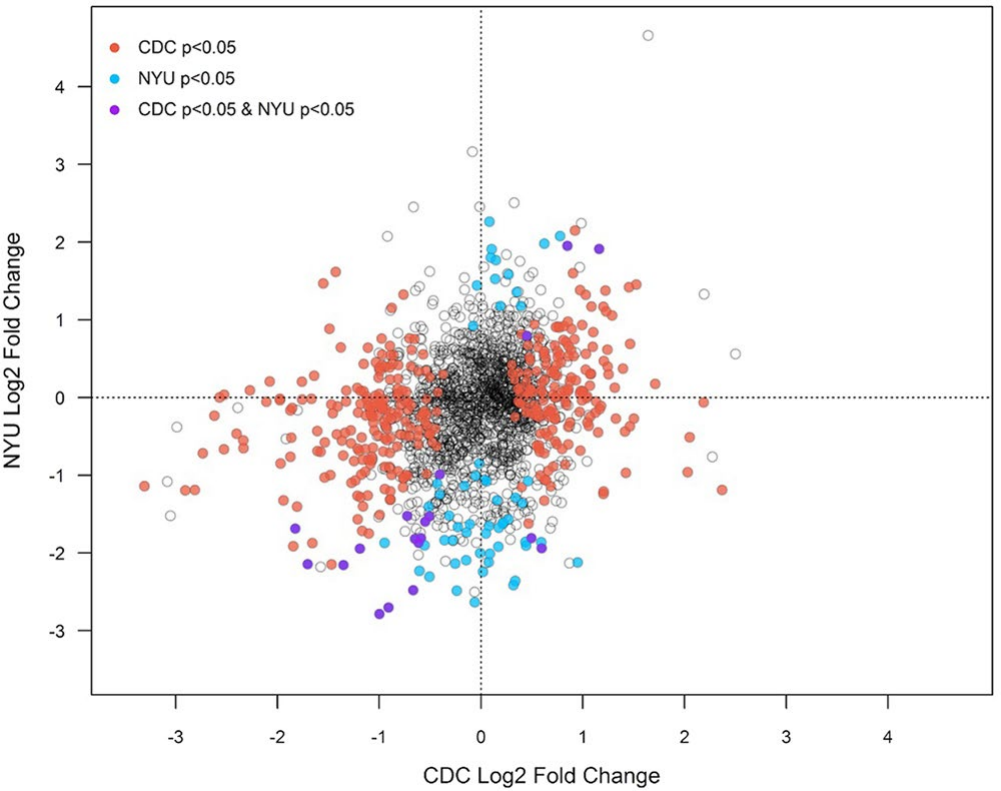
Scatterplot of obesity-associated OTUs in the CDC and NYU studies. All of the OTUs tested (1,825) are plotted by their log2 fold changes (obese vs. healthy-weight) in the CDC and NYU studies. OTUs represented by black open circles were not significantly associated with obesity in either study. Red, blue, and purple circles represent OTUs associated with obesity (p < 0.05) in the CDC study only, NYU study only, or in both studies, respectively. OTU models with extreme outliers (maximum Cook’s distance >15) are not colored in the plot. R^2^ = 4.8%.

We observed consistencies in taxa associated globally with BMI category (q < 0.05) between the CDC and NYU studies (Supplemental Table 7; Fig. 2), despite the much smaller sample size of the NYU study. In pairwise comparisons in both studies, obese participants had increased abundance of Bacilli (Streptococcaceae and Lactobacillaceae families) and Gammaproteobacteria, and decreased abundance of Christensenellaceae, compared to healthy-weight participants (p_Holm_ < 0.05). At the OTU level, we observed substantially more OTUs associated globally with BMI category (q < 0.05) in the CDC study than in the NYU study, likely due to the substantially smaller sample size of the NYU study, and the large number of tests. We therefore explored similarities between the studies at the OTU level using nominal p-values. 17 OTUs were associated with obesity (p < 0.05) in the same direction in both studies, while only 2 OTUs were associated with obesity (p < 0.05) in the opposite direction between the studies (Supplemental Table 8; Fig. 5). The OTUs overlapping across the studies in significance and direction included Gemellaceae, *Streptococcus*, and *Blautia* OTUs (increased in the obese), and *Parabacteroides*, Clostridiaceae, Lachnospiraceae, Ruminococcaceae, Clostridiales, and *Oscillospira* OTUs (decreased in the obese).

### Microbiome-based classification of obesity

**Figure 6:**
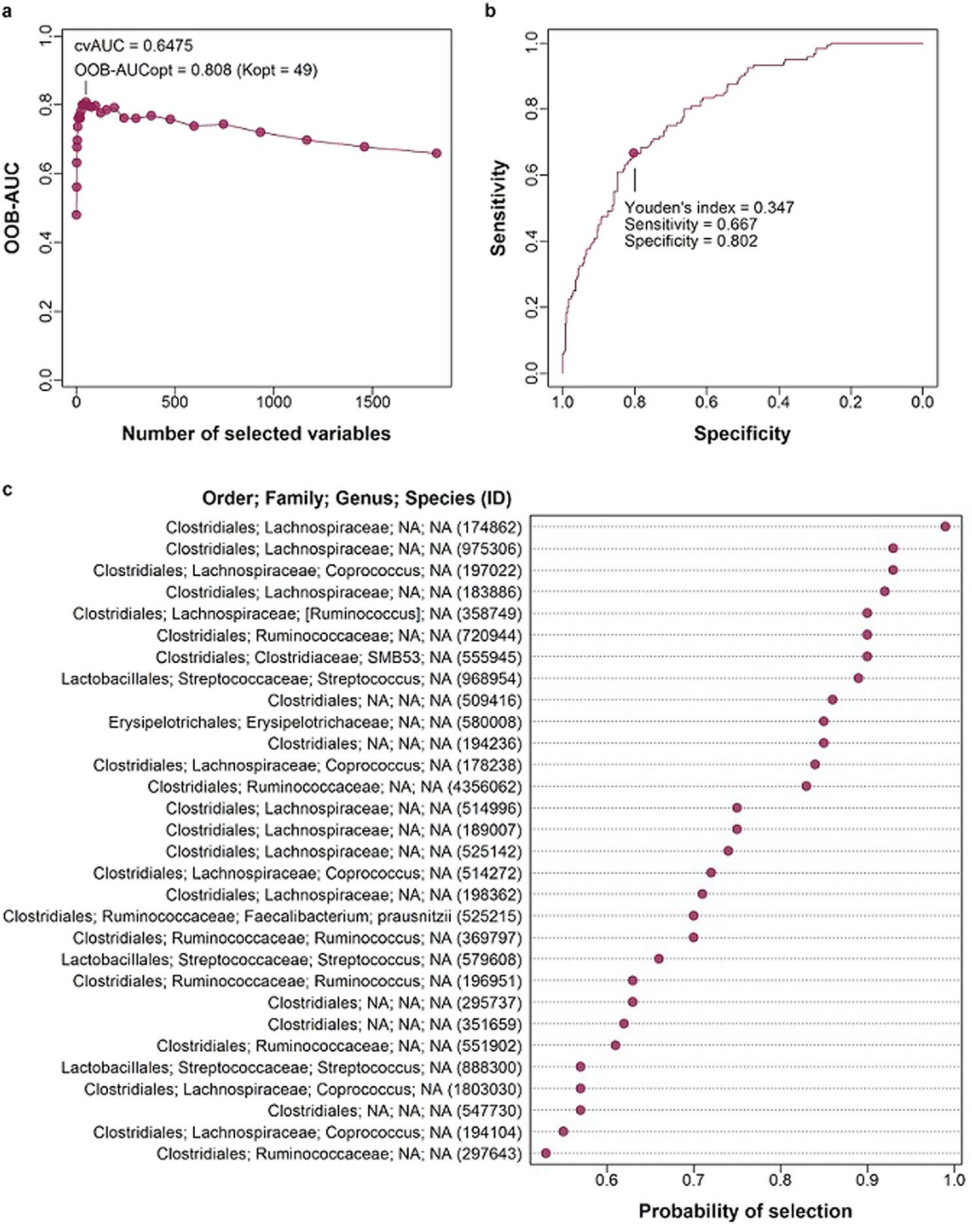
Random forest model of the training data set (CDC study). A random forest model was generated based on 1,825 DESeq2-normalized OTUs in the training data set (CDC study) using the AUCRF R package. (**a**) The optimal random forest model of 49 OTUs was selected by optimizing the area under the receiver operating characteristic (ROC) curve (AUC) of the random forest (optimal AUC = 0.81); the mean AUC of repeated (20 times) 5-fold cross-validations of the random forest model was 0.65. (**b**) ROC curve of the optimal random forest model, highlighting Youden’s index (probability at maximum sum of sensitivity and specificity). (**c**) Top 30 OTUs with highest probability of selection in repeated cross-validation of the optimal random forest model.

We generated a random forest model based on 1,825 OTUs in the CDC study (training set) to predict obesity in the NYU and Baxter *et al*.^26^ studies (testing sets). We used the area under the curve-random forest (AUC-RF) algorithm to perform a backward elimination process based on the initial ranking of OTUs in a random forest model; this algorithm identifies the optimal random forest model (and optimal set of predictive OTUs) as the model with the highest AUC. Our optimal model included 49 OTUs and had an AUC of 0.81 (Fig. 6). We then performed repeated cross-validation of the AUC-RF process to more accurately determine the model’s predictive accuracy; the mean AUC from repeated cross-validation was 0.65. We used the Youden’s index of the ROC curve as the probability threshold above which a subject was classified as obese in the testing sets. The accuracy of the model in correctly classifying subjects as obese or non-obese when applied to the NYU and Baxter *et al*. testing sets was 0.72 and 0.68, respectively.

## Discussion

In this large study of older American adults, we observed that obesity was associated with reduced gut microbial richness and alterations in overall gut microbial composition. These findings point to a possible effect of gut microbial composition on energy balance or storage. The homogeneity of our results in two independent study populations, and the good accuracy of obesity classification with a microbiome-based machine learning model, reveals an emerging taxonomic signature of obesity which may have implications for obesity prevention and treatment.

Several mechanisms have been hypothesized through which gut bacteria may affect host energy balance or storage. The “energy harvest” hypothesis posits that bacteria contribute to obesity by extracting energy from otherwise indigestible dietary fiber, through production of digestible short-chain fatty acids (SCFAs)^9^. The “metabolic endotoxemia” hypothesis posits that plasma lipopolysaccharide (LPS, or endotoxin) derived from the cell wall of Gram-negative bacteria elicits low-grade inflammation, promoting adiposity^27,28^. A final broad category of mechanisms is that of microbial metabolites or products modulating energy balance^7^. Notably, SCFAs, in addition to being energy sources to the host, are important signaling molecules with beneficial effects for host energy metabolism^29^, and protect against diet-induced obesity in animal models^30,31^. Other bacterial metabolites, such as methane^32^ and secondary bile acids^33^, may also modulate host energy balance. Here, we observed many taxonomic composition alterations associated with obesity. Whether and by what mechanism these bacterial groups impact obesity remains unclear, but we discuss some potential mechanisms in relation to our findings below.

Decreases in putative SCFA-producing bacteria in the obese may lend support to the hypothesis that SCFAs beneficially modulate host energy metabolism. The Christensenellaceae family is known to produce SCFAs, primarily acetate and butyrate^34^, and was identified as the most heritable taxon in a study of 416 twin pairs; in that study, Christensenellaceae, Dehalobacteriaceae, SHA-98, Methanobacteriaceae, RF39, and *Oscillospira* were depleted in obese subjects compared to healthy-weight^35^, much in agreement with our findings. Higher Christensenellaceae abundance in mice that received human fecal transplants was correlated with reduced weight gain, and transplant of obese donor stool amended with *Christensenella minuta* to recipient mice led to reduced adiposity^35^. Findings of depleted Christensenellaceae in obese individuals have since been replicated in other large studies^21,36^, indicating that Christensenellaceae may be important for promoting leanness. *Oscillospira* has also been suggested to promote human leanness; it was enriched in healthy-weight subjects in several human studies^36,37^, and may contribute to leanness by degrading host glycans and producing SCFAs^37^. We also observed that other Clostridiales OTUs (Ruminococcaceae, Lachnospiraceae, and unclassified families) were depleted in the obese; although functions of these bacteria are unknown, many members of these families produce SCFAs^25^. An important caveat is that multitudes of gut bacteria produce SCFAs, making it unclear whether this mechanism is actually responsible for patterns observed. Our inferred metagenome analysis, however, revealed that the KEGG pathway related to the SCFA butyrate (“butanoate metabolism”) was marginally depleted in obese compared to healthy-weight participants, supporting the beneficial SCFA hypothesis.

Increases in Enterobacteriaceae in the obese may lend support to the “metabolic endotoxemia” hypothesis, as LPS from Enterobacteriaceae exhibits high endotoxin activity^27^; however the “LPS biosynthesis” pathway was not associated with obesity in our inferred metagenome analysis. Enterobacteriaceae species were also associated with obesity in other studies^38,39^, and have been shown to decrease following weight-loss interventions^40,41^.

Synthesis of secondary bile acids and methane represent other potential mechanisms by which gut microbiota may modulate host energy balance. In our inferred metagenome analysis, we observed that “secondary bile acid biosynthesis” was marginally enriched in obese compared to healthy-weight participants, while “methane metabolism” was not associated with obesity. Some species in *Clostridium* and *Eubacterium* generate secondary bile acids^42^, which may modulate adiposity via farnesoid X receptor (FXR) or Takeda G-protein-coupled receptor 5 (TGR5) signaling^33,43^. Methanogens may promote adiposity via conversion of hydrogen to methane gas^32,44^, and have previously been associated with leanness by other studies^16,35,45^, or, in contrast, with obesity^46–48^. More research is needed in human populations to elucidate the roles of secondary bile acids and methane in obesity.

We also identified that the “ALA metabolism” KEGG pathway was enriched in obese compared to healthy-weight participants. ALA is a type of n-3 polyunsaturated fatty acid, which may be metabolized to conjugated linolenic acids by gut microbiota^49^; conjugated linolenic acids were shown to have anti-adipogenic properties in several studies^50^, in contradiction with this observed result.

We observed reduced microbial diversity in the obese, particularly for women. Obesity-related reductions in microbial diversity have been reported previously^15,21,36,39,51^, though not by all^22,52^. One study related the reduction in diversity to “abnormal energy input” in obesity^15^. Individuals with low microbial gene richness are more likely to be obese and have poorer metabolic health^53^. Additionally, a weight-loss intervention was less effective at improving inflammatory markers in those with low microbial gene richness^54^. Therefore, low microbial diversity may be a further factor conferring susceptibility to obesity. The reason for the sex difference in our microbiome diversity result is unclear; a possible mechanism may lie in the effect of sex hormones on the gut microbiota^55^, however replication of the result in other studies is warranted.

The potential for manipulation of gut microbiota has generated interest in identifying a taxonomic signature for obesity that is responsible for the obesogenic mechanisms detailed above. Animal studies and some small human studies have demonstrated that the obese microbiome is characterized by a phylum-level signature of increased Firmicutes and decreased Bacteroidetes^8,9,12,15^. However, larger human studies have failed to replicate this signature^21–23^, including the current study. It is possible that in humans, the taxonomic signature of obesity exists on a finer species (OTU) level, rather than at phylum level. Further, due to large between-person and between-population variability in the gut microbiome, large sample sizes are likely needed to detect such a signature. This signature may differ by population factors such as age, race, and geography. We have observed consistency of findings between our two independent study populations, which both consisted of older, mostly white Americans, suggesting that a taxonomic signature of obesity can be identified within homogeneous populations. In support of this, a recent meta-analysis robustly replicated eight obesity-associated OTUs across three large population-based cohorts of European descent^36^. Additionally, we observed good accuracy (~70%) of obesity classification by a microbiome-based random forest model, trained on one study and tested on two studies with similar population characteristics to the training set. An analysis of 10 published datasets by another group observed overall poor accuracy of random forest models trained on one dataset and tested on the other nine (median accuracy 33–65% for 10 models)^24^. However these datasets differed substantially on population characteristics such as age, race, and geography, which all may impact model performance; here we have focused on homogeneous populations, assuming there is no universal taxonomic signature of obesity across all populations. Additionally, the authors used genus-level information to develop the models, whereas here we used OTU-level information, which could also impact model performance. Regardless of whether high accuracy of obesity classification can be achieved with machine learning, it remains possible that specific taxa play a mechanistic role in obesity.

Strengths of this study include the large sample size, control of potential confounders, comprehensive bacterial profiling, and availability of dietary data in a subset of participants. The effect of diet on gut microbial composition has been demonstrated previously^56–59^; due to effects of diet on both microbiota and BMI, it is difficult to tease apart potential microbial contributions to obesity from effects of diet on microbiota. Here, adjustment for dietary factors did not impact the association of obesity with microbial composition. Although power of this analysis was limited due to the small subset with dietary information (n = 171) and measurement error inherent in food frequency questionnaires^60^, it suggests a relationship between microbial composition and obesity independent of diet. Our study also has several limitations. The cross-sectional design does not allow us to establish temporality or causality of the microbiome-obesity relationship. Additionally, due to the older age and mostly white study population (96% 50 and over; 94% white), findings may not be generalizable to younger or more diverse populations. We also lacked antibiotic usage information in the CDC study which did not allow us to exclude individuals taking antibiotics, and we lacked dietary and exercise data in the CDC study which did not allow us to adjust for these potential confounders in the full study population. Finally, lack of shotgun-sequenced metagenome data did not allow us to actually characterize metagenomic functions.

In summary, in this large study of older American adults, we observed a significant relationship between the gut microbiome and obesity. The taxa identified may open new avenues for experimental research on causal microbial agents of obesity. Additional large-scale studies are warranted in humans to confirm a taxonomic signature of obesity (in a variety of populations, as the signature may vary by age, race, and geography). From there, interventions in animals and humans can identify obesity-promoting bacteria or lean-promoting bacteria, and the mechanisms of their action. Looking forward, precision medicine approaches based on an individual’s microbiome may eventually be used to effectively treat or prevent obesity.

## Methods

### Study population

We included data from two independent study populations based at colonoscopy clinics: the Centers for Disease Control and Prevention Study of In-home Tests for Colorectal Cancer (CDC study)^61^, and the New York University Human Microbiome and Colorectal Tumor study (NYU study)^62^ (Supplemental Fig. 4). The CDC study was approved by the institutional review boards of University of Minnesota and the CDC, and the NYU study by the institutional review board of NYU School of Medicine. Methods were carried out in accordance with relevant guidelines and regulations, and all participants provided informed consent.

The CDC study contributed 451 subjects at University of Minnesota/Minnesota Gastroenterology (12/2012-7/2014). Eligible participants were 50–75 years old, scheduled to have a colonoscopy for routine screening, able to read English, and not currently taking anticoagulants. Additionally, participants must not have had >1 episode of rectal bleeding in the last six months, a positive FOBT in the past twelve months, a colonoscopy in the past 5 years, a personal history of colorectal cancer, polyps, or inflammatory bowel disease, or a personal or family history of familial adenomatous polyposis or hereditary nonpolyposis colorectal cancer. We excluded participants that withdrew (n = 17), subjects for whom sequencing failed (n = 4), subjects missing BMI (n = 3), and underweight subjects (BMI < 18.5 kg/m^2^; n = 4), resulting in 423 subjects.

The NYU study enrolled 239 participants from Kips Bay Endoscopy Center in New York City (6/2012-8/2014). Eligible participants were 18 years or older (range: 29–86), recently underwent colonoscopy, able to read English, and not on long-term antibiotics. We excluded participants missing colonoscopy reports (n = 2), missing BMI (n = 9), or underweight (n = 1), and further excluded participants with rectal bleeding (n = 18) or with personal history of colorectal cancer (n = 10), inflammatory bowel disease (n = 22), anastomosis (n = 6), or familial adenomatous polyposis (n = 1), in order to conform the NYU study to the CDC study; exclusion based on these non-mutually exclusive criteria resulted in 176 subjects.

### Stool samples

Subjects collected stool onto Beckman Coulter Hemoccult II SENSA® cards (Beckman Coulter, CA) at home. This method produces reproducible and accurate 16S rRNA gene-derived microbiota data^63,64^, and exhibits stability at room temperature up to 8 weeks^65^. CDC samples were mailed to a laboratory for fecal occult blood testing within several days of stool collection; this testing does not impact microbiota composition^62,63^. After testing, CDC samples were refrigerated at 4 °C until shipment to NYU, and upon arrival were stored at −80 °C (range: 7–183 days from sample collection to receipt by NYU). NYU samples were mailed directly to NYU following at-home collection and stored immediately at −80 °C.

### Microbiome assay

DNA was extracted from stool using the PowerLyzer PowerSoil Kit (Mo Bio Laboratory Inc., CA) following manufacturer’s protocol, as described previously^62^. Barcoded amplicons were generated covering the V4 region of the 16S rRNA gene using the F515/R806 primer pair^66^. The PCR reaction, using FastStart High Fidelity PCR system, dNTP pack (Roche, IN), was run as follows: initial denaturing at 94 °C for 3 min, followed by 25 cycles of 94 °C for 15 s, 52 °C for 45 s and 72 °C for 1 min, and a final extension at 72 °C for 8 min. PCR products were purified using Agencourt AMPure XP (Beckman Coulter Life Sciences, IN) and quantified using the Agilent 4200 TapeStation (Agilent Technologies, CA). Amplicon libraries were pooled at equal molar concentrations and sequenced on Illumina MiSeq with a 300-cycle (2 × 151 bp) kit.

### Sequence read processing

Forward and reverse reads were joined using *join_paired_ends.py* in QIIME with default parameters^67^. Sequences were demultiplexed, and poor-quality sequences excluded, using default parameters of QIIME script *split_libraries*_ *fastq.py*; median sequence length was 253 base pairs. Chimeric sequences were excluded using USEARCH 6.1, with the “gold” reference database (Broad Institute Microbiome Utilities microbiomeutil-r20110519). Sequence reads were clustered into operational taxonomic units (OTUs) against the Greengenes 13_8 reference sequence collection, using QIIME *pick_closed_reference_otus.py* script (results were highly similar using *de novo* OTU picking, data not shown). The final dataset of 599 participants included 15,098,120 sequence reads (mean ± SD: 25,206 ± 15,616 reads/sample) and 8,902 OTUs. Quality control data showing excellent reproducibility for this data has been published previously^62^.

### Covariates

Only limited demographic information (age, sex, BMI, race) was collected during CDC study enrollment. The NYU study collected more extensive information (e.g. data on exercise, smoking, health history, and dental health) and food frequency questionnaires. The food frequency questionnaire used in the NYU study was the 137-item DQX from the National Cancer Institute Prostate, Lung, Colorectal, and Ovarian Cancer screening trial (PLCO), available at https://biometry.nci.nih.gov/cdas/datasets/plco/97/. Nutrient variables were calculated following the PLCO protocol; briefly, the frequency for each line item was multiplied by a nutrient amount (derived from the USDA CSFII database) which was dependent on the gender of the subject as well as the response to serving size, when applicable. Healthy-weight was defined as BMI ≥ 18.5 and <25 kg/m^2^, overweight as BMI ≥ 25 and <30 kg/m^2^, and obese as BMI ≥ 30 kg/m^2^. Colorectal polyps were identified at colonoscopy and confirmed by pathology; cases were defined as those with ≥1 polyp of non-normal histology, or those with history of polyps.

### α-diversity

α-diversity (within-subject species diversity) was assessed using richness, Shannon diversity index, and evenness, calculated in 100 iterations for rarefied OTU tables (minimum: 50 reads/sample, maximum: 1,490 reads/sample [lowest participant sequencing depth]) using QIIME script *alpha_rarefaction.py*. We examined whether α-diversity (at 1,490 sequence reads/sample) differed across BMI categories using linear regression, adjusting for age, sex, polyp status, and study. Statistical significance of the global BMI category variable was determined using an F-test comparing the full vs. reduced model (i.e. without BMI category). P-values for the two pairwise comparisons of interest (obese vs. healthy-weight and overweight vs. healthy-weight) were adjusted with the Holm method^68^.

### β-diversity

β-diversity (between-subject species diversity) was assessed using the weighted UniFrac distance^69^. Principal coordinate analysis (PCoA)^70^ and partial constrained analysis of principal coordinates (CAP)^71^ were used to visually explore the relationship between BMI and overall bacterial composition. In partial CAP analysis, BMI category was the constraining variable, and sex, age, polyp status, and study were conditioning variables. Permutational multivariate analysis of variance (PERMANOVA)^72^ was used to examine statistically whether overall bacterial composition differed by BMI category, adjusting for age, sex, polyp status, and study. Statistical significance was determined as described above for α-diversity.

### Differential abundance testing

To examine differences in abundance of bacterial taxa across BMI categories we used negative binomial generalized linear models (DESeq2)^73^. This method models raw counts with a negative binomial distribution, adjusting internally for “size factors” which normalize for differences in sequencing depth between samples. The raw counts of 8,902 OTUs were agglomerated to 14 phyla, 30 classes, 56 orders, 115 families, 302 genera, and 413 species. Prior to analysis, we filtered the data to include only taxa with ≥2 sequence reads in ≥5% of participants (30 participants), resulting in inclusion of 11 phyla, 20 classes, 25 orders, 52 families, 100 genera, 133 species, and 1,825 OTUs. DESeq2 models were adjusted for age, sex, polyp status, and study. DESeq2 default outlier replacement, independent filtering of low-count taxa, and filtering of count outliers were turned off. We used likelihood-ratio tests (LRT) to determine statistical significance of the global BMI category variable in DESeq2 models; we adjusted the p-values for taxa at each level (i.e. class, genus) for the false discovery rate (FDR)^74^, with models with maximum Cook’s distance >15 removed prior to p-value adjustment. For models that were significant (LRT FDR-adjusted p-value [q-value] < 0.05), Wald test p-values for the two pairwise comparisons of interest (obese vs. healthy-weight and overweight vs. healthy weight) were adjusted with the Holm method^68^. This methodology controls the mixed directional FDR^75^.

### Inferred metagenomes

PiCRUST^76^ was used to infer metagenomic content from 16S rRNA gene-based microbial compositions. The 5,753 observed KEGG^77^ gene orthologs were grouped into 276 KEGG pathways. We filtered the data to include only pathways with ≥2 reads in ≥30 participants, and removed unclassified pathways and pathways related to “Human Diseases” or “Organismal Systems”, resulting in inclusion of 185 pathways. We used DESeq2 (as described above) to test differences in pathway abundance across BMI categories. Statistical significance was determined as described above for differential abundance testing. We considered nominal p-values for *a priori* pathways of interest, and q-values for other pathways. We used partial Spearman’s correlations to examine associations between taxa and pathways, adjusting for age, sex, study, and polyp status. We also explored OTU contributions to *a priori* KEGG orthologs of interest using PiCRUST script *metagenome_contributions.py*.

### Random forest machine learning

We used a random forest model based on the CDC study (training set) to classify individuals in the NYU study and another human study^26^ (testing sets) as obese (BMI ≥ 30 kg/m^2^) or non-obese (BMI < 30 kg/m^2^). We chose the Baxter *et al*. study due to its similarity with our study, as it was also colonoscopy-based and comprised of older, mostly white Americans. The Baxter *et al*. data was downloaded from the NCBI Sequence Read Archive (SRP062005) and processed identically to our data (see “Sequence read processing” in Methods), to facilitate comparison with our studies. After excluding participants with cancer, the Baxter *et al*. data comprised 402 subjects (age mean ± SD = 59.5 ± 11.7, 91% white, 50% men). The random forest model for the training set was generated using the AUCRF R package^78^, which performs variable selection based on optimizing the area under the receiver operating characteristic (ROC) curve (AUC) of the random forest. DESeq2-normalized counts of 1,825 OTUs were used in variable selection. We performed repeated (20 times) 5-fold cross-validation of the random forest model. The probability threshold above which a subject was classified as obese in the testing sets was based on Youden’s index (probability at maximum sum of sensitivity and specificity) of the ROC curve of the training set model. Accuracy was calculated as (true positives + true negatives)/(total subjects).

### Diet and exercise sensitivity analysis

In the NYU study, data on diet (e.g. total energy, fiber, protein, fat intake) and exercise were available, and we checked whether adjusting for these variables in the NYU study influenced our overall (α- and β-) diversity results. Models with fiber, protein, or fat intake were adjusted for total energy. Those with unrealistic total energy intake (<500 or >4000 kcal/day; n = 3) and those leaving blank >50% of the items on the 137-item food frequency questionnaire (n = 2) were considered missing and excluded from the dietary analysis. Those missing exercise data (n = 1) were excluded from the exercise analysis.

### Data availability statement

The datasets analyzed during the current study are available in the dbGaP repository (accession phs001381.v1.p1).

## Electronic supplementary material

Supplementary Information
