# Supplementary material for: A taxonomic signature of obesity in a large study of American adults
Source: Sci Rep. 2018 Jun 27;8:9749. doi: 10.1038/s41598-018-28126-1 (PMC6021409; doi:10.1038/s41598-018-28126-1)
Supplement: Supplementary file 1 — Supplementary Information [file 41598_2018_28126_MOESM1_ESM.pdf]

SUPPLEMENTAL MATERIAL

A taxonomic signature of obesity in a large study of American adults

Brandilyn A. Peters,[1] Jean A. Shapiro,[2] Timothy R. Church,[3] George Miller,[4,5,6] Chau Trinh-Shevrin,[1,6] Elizabeth Yuen,[7] Charles Friedlander,[7] Richard B. Hayes,[1,6] Jiyoung Ahn[1,6]

[1]Department of Population Health, New York University School of Medicine, New York, NY, USA

[2]Division of Cancer Prevention and Control, Centers for Disease Control and Prevention, Atlanta, GA, USA

[3]Division of Environmental Health Sciences, School of Public Health, University of Minnesota, Minneapolis, MN, USA

[4]Departments of Surgery and [5]Cell Biology, New York University School of Medicine, New York, NY, USA

[6]NYU Perlmutter Cancer Center, New York University School of Medicine, New York, NY, USA

[7]Kips Bay Endoscopy Center, New York, NY, USA

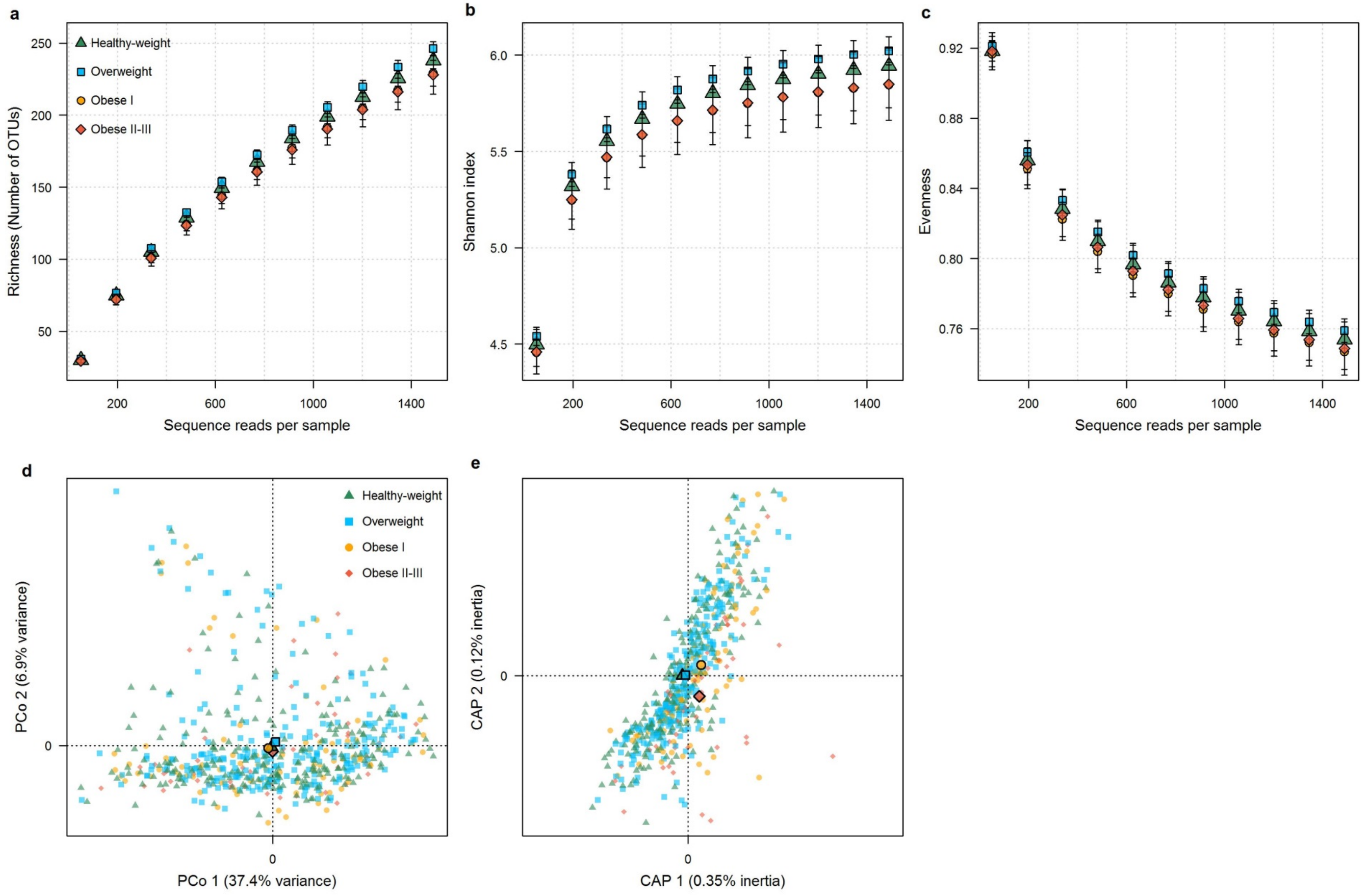

Supplemental Figure 1. (a, b, c) Richness, Shannon diversity index, and Evenness rarefaction curves in healthy-weight, overweight, obese class I, and obese class II-III participants. Rarefaction curves were estimated by taking the mean of the α-diversity indices averaged for each participant over 100 iterations at each rarefaction sequencing depth. (d) Principal coordinate analysis of the weighted UniFrac distances. Shapes outlined in black represent centroids for healthy-weight, overweight, and obese participants. (e) Partial constrained analysis of principal coordinates (CAP) based on the weighted UniFrac distance. BMI category was the constraining variable, and sex, age, polyp status, and study were conditioning variables.

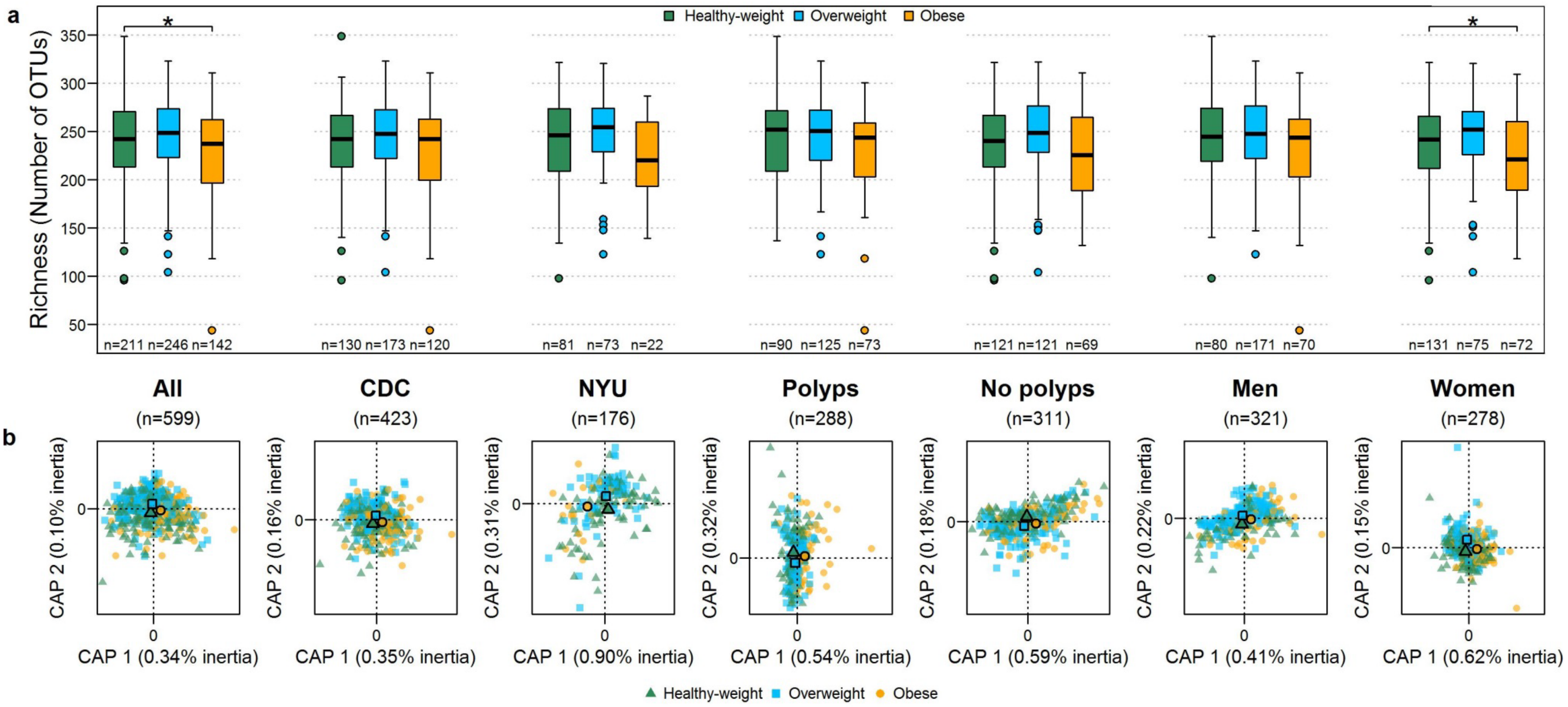

Supplemental Figure 2. (a) Boxplots of richness (number of OTUs) in relation to BMI in subgroups by study, polyp status, and sex. P-values were obtained from linear regression models with richness at 1,490 sequence reads per sample as the outcome, and BMI category as the main predictor. Study-stratified models were adjusted for age, sex, and polyp status; polyp-stratified models were adjusted for age, sex, and study; sex-stratified models were adjusted for age, study, and polyp status. (b) Partial CAP of the weighted UniFrac distance within sub-groups, with BMI category as the constraining variable, and conditioning variables as listed for (a). Shapes outlined in black represent centroids for healthy-weight, overweight, and obese participants.
* $p < 0.05$

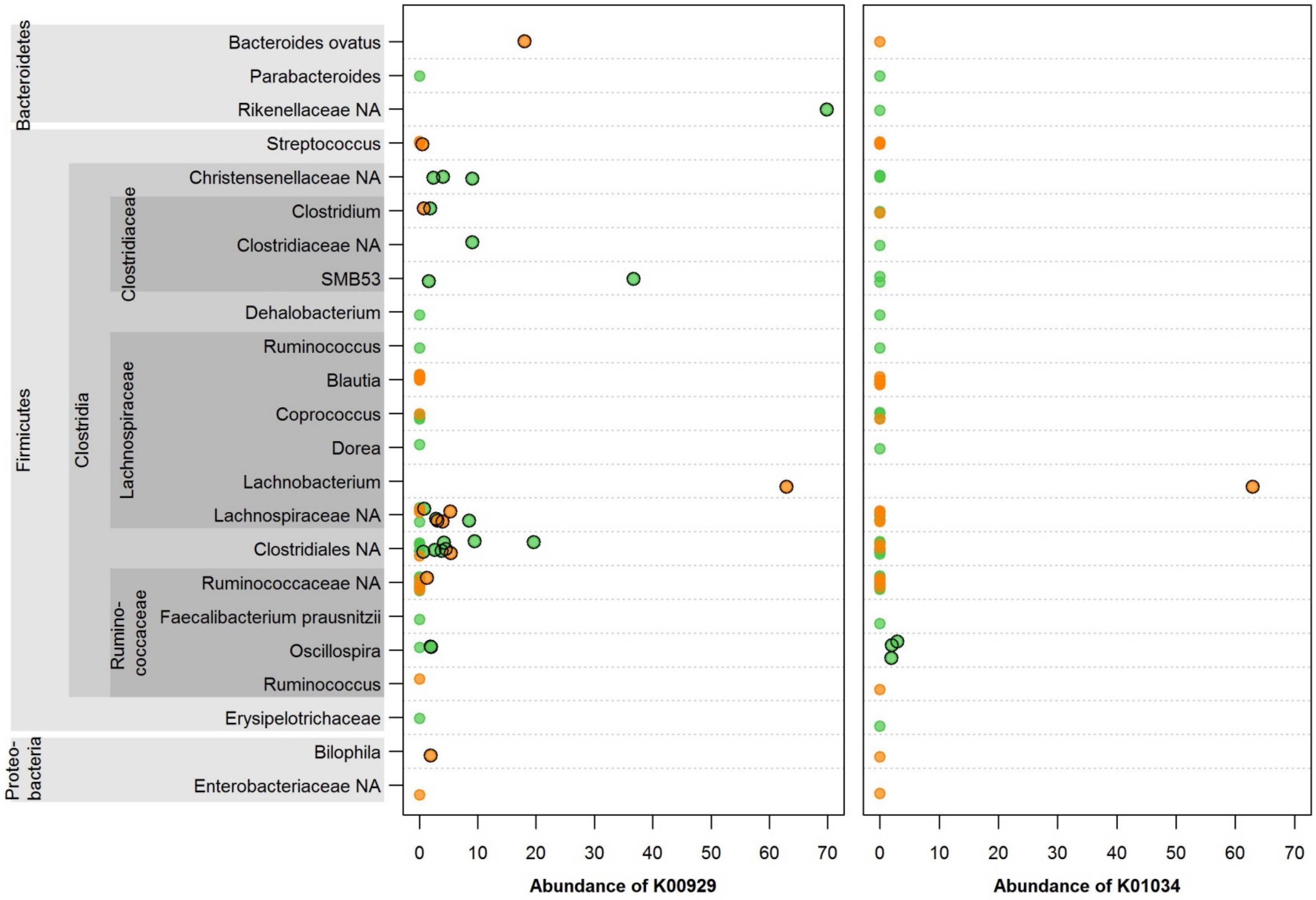

Supplemental Figure 3. Mean contributions of OTUs associated with obesity to abundance of KEGG orthologs for butyrate synthesis (K00929: butyrate kinase, K01034: acetate CoA/acetoacetate CoA-transferase alpha subunit). Abundance contribution was averaged over all participants for each OTU. Only OTUs associated with obesity (LRT q<0.05 and p_{Holm}<0.05) are plotted; green points represent OTUs depleted in the obese, and orange points represent OTUs enriched in the obese. Circles with black outlines indicate OTUs with non-zero contribution to the abundance of the KEGG ortholog. Another butyrate synthesis KEGG ortholog, K01035 was excluded from the plot since none of the obesity-associated OTUs contributed to its abundance.

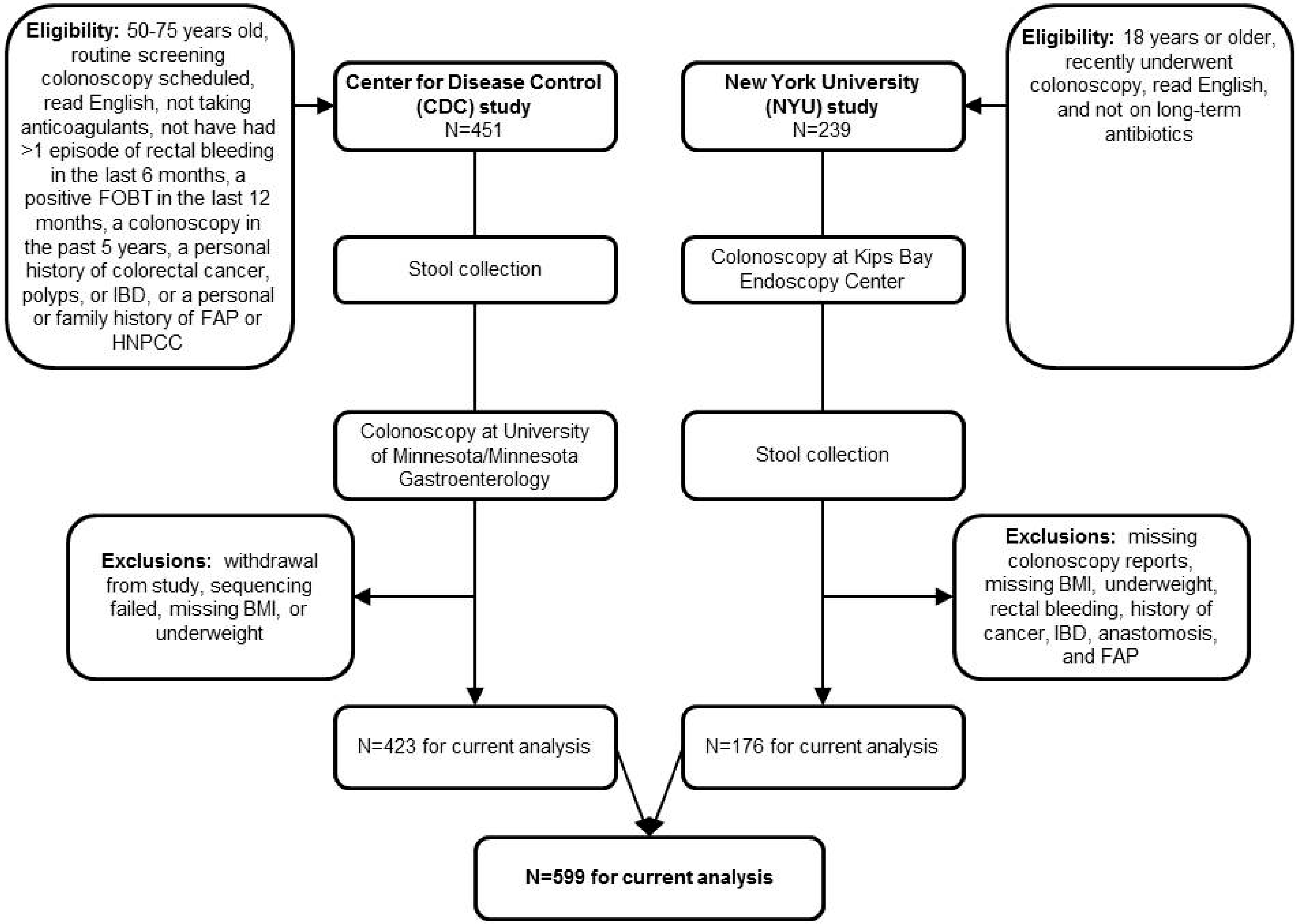

Supplemental Figure 4. Flow chart of the study design.

Supplemental Table 1. Obesity and overweight in relation to α- and β-diversity.

	F-test (3-cat.)[b]	F-test (4-cat.)[b]	Obese vs. healthy-weight		Overweight vs. healthy-weight		Obese I vs. healthy-weight		Obese II-III vs. healthy-weight	
	p	p	β (se)	p (p_{Holm})[c]	β (se)	p (p_{Holm})[c]	β (se)	p (p_{Holm})[d]	β (se)	p (p_{Holm})[d]
Richness[a]	0.002	0.005	-9.87 (4.68)	0.04 (0.08)	6.47 (4.12)	0.12 (0.12)	-9.72 (5.40)	0.07 (0.21)	-10.13 (6.63)	0.13 (0.24)
Shannon index[a]	0.03	0.07	-0.11 (0.07)	0.11 (0.22)	0.06 (0.06)	0.28 (0.28)	-0.11 (0.08)	0.14 (0.42)	-0.10 (0.10)	0.29 (0.56)
Evenness[a]	0.14	0.26	-0.01 (0.01)	0.22 (0.44)	0.00 (0.01)	0.43 (0.44)	-0.01 (0.01)	0.22 (0.66)	-0.01 (0.01)	0.51 (0.86)
	p	p	R^2 (%)	p (p_{Holm})[c]	R^2 (%)	p (p_{Holm})[c]	R^2 (%)	p (p_{Holm})[d]	R^2 (%)	p (p_{Holm})[d]
Weighted UniFrac[e]	0.14	0.28	0.37	0.037 (0.074)	0.10	0.639 (0.639)	0.32	0.071 (0.213)	0.22	0.194 (0.388)

[a]Parameters are from linear regression models with specified α-diversity metric (averged over 100 iterations of rarefied OTU table at 1,490 sequence reads/sample) as outcome. All models were adjusted for age, sex, polyp status, and study.
[b]Test of global BMI category variable; 3-category indicates healthy-weight, overweight, and obese groups; 4-category indicates healthy-weight, overweight, obese I, and obese II-III groups.
[c]p-values were adjusted with the Holm method for two pairwise comparisons: obese vs. healthy-weight and overweight vs. healthy-weight.
[d]p-values were adjusted by with the Holm method for three pairwise comparisons: obese I vs. healthy-weight, obese II-III vs. healthy-weight, and overweight vs. healthy-weight.
[e]Parameters are from permutational MANOVA of weighted UniFrac distance using 'adonis' function (Vegan package, R); adjustment factors of age, sex, polyp status, and study were included in the model first before BMI category.

Supplemental Table 2. Obesity in relation to α- and β-diversity in sub-groups by study, polyp status, and sex.

	CDC study (n=423)		NYU study (n=176)		Polyps (n=288)		No polyps (n=311)		Men (n=321)		Women (n=278)	
	β (se)	p	β (se)	p	β (se)	p	β (se)	p	β (se)	p	β (se)	p
Richness[a,c]	-9.13 (5.31)	0.09	-16.86 (10.58)	0.11	-7.44 (6.58)	0.26	-12.23 (6.71)	0.07	-4.99 (6.95)	0.47	-14.41 (6.41)	0.03
Shannon index[a,c]	-0.12 (0.08)	0.11	-0.13 (0.15)	0.39	-0.08 (0.09)	0.4	-0.14 (0.10)	0.15	-0.07 (0.10)	0.51	-0.15 (0.09)	0.09
Evenness[a,c]	-0.01 (0.01)	0.16	-0.01 (0.01)	0.67	0.00 (0.01)	0.54	-0.01 (0.01)	0.26	0.00 (0.01)	0.59	-0.01 (0.01)	0.19
	R^2 (%)	p	R^2 (%)	p	R^2 (%)	p	R^2 (%)	p	R^2 (%)	p	R^2 (%)	p
Weighted UniFrac[b,c]	0.38	0.15	0.95	0.132	0.56	0.125	0.46	0.189	0.39	0.229	0.65	0.106

[a]Parameters are for obese vs. healthy-weight comparison in linear regression models with specified α-diversity metric (averaged over 100 iterations of rarefied OTU table at 1,490 sequence reads/sample) as outcome.
[b]Parameters are for obese vs. healthy-weight comparison in permutational MANOVA of UniFrac distances using 'adonis' function (Vegan package, R); adjustment factors of age, sex, polyp status, and study were included in the model first before BMI category.
[c]Study-stratified models were adjusted for age, sex, and polyp status; polyp-stratified models were adjusted for age, sex, and study; sex-stratified models were adjusted for age, study, and polyp status.

Supplemental Table 3. Obesity in relation to α- and β-diversity after adjustment for dietary factors or exercise in the NYU study.

	No diet adjustment[a]		Total energy adjustment[a,b]		Fiber adjustment[a,c]		Fat adjustment[a,c]		Protein adjustment[a,c]		No exercise adjustment[d]		Exercise adjustment[d,e]	
	β (se)	p	β (se)	p	β (se)	p	β (se)	p	β (se)	p	β (se)	p	β (se)	p
Richness[f]	-15.18 (10.86)	0.16	-15.91 (10.77)	0.14	-15.47 (10.79)	0.15	-14.61 (10.83)	0.18	-15.72 (10.95)	0.15	-19.45 (10.75)	0.07	-24.26 (11.53)	0.04
Shannon index[f]	-0.10 (0.15)	0.52	-0.11 (0.15)	0.48	-0.11 (0.15)	0.49	-0.09 (0.15)	0.57	-0.10 (0.16)	0.52	-0.16 (0.15)	0.3	-0.24 (0.16)	0.14
Evenness[f]	0.00 (0.01)	0.81	0.00 (0.01)	0.78	0.00 (0.01)	0.78	0.00 (0.01)	0.89	0.00 (0.01)	0.84	-0.01 (0.01)	0.56	-0.02 (0.01)	0.29
	R^2 (%)	p	R^2 (%)	p	R^2 (%)	p	R^2 (%)	p	R^2 (%)	p	R^2 (%)	p	R^2 (%)	p
Weighted UniFrac[g]	0.86	0.155	0.88	0.164	0.86	0.152	0.83	0.173	0.83	0.184	1.02	0.101	1.10	0.097

[a]Models were examined in n=171 NYU participants with diet data; all models were adjusted for age, sex, and polyp status; model parameters shown are for obese vs. healthy-weight comparison.
[b]Total energy intake (kcal) was added to the model.
[c]Fiber, fat, or protein was added to the model; these models also adjust for total energy intake.
[d]Models were examined in n=175 NYU participants with exercise data; all models were adjusted for age, sex, and polyp status; model parameters shown are for obese vs. healthy-weight comparison.
[e]Exercise was added to the model.
[f]These α-diversity indices were the outcomes in linear regression models.
[g]Permutational MANOVA of the UniFrac distances using 'adonis' function (Vegan package, R); all adjustment factors were included in the model first before BMI category.

Supplemental Table 4. Differentially abundant taxa between obese and healthy-weight participants, and overweight and healthy-weight participants, as detected by the DESeq function in the DESeq2 package. Models were adjusted for age, sex, polyp status, and study. All taxa with an LRT FDR-adjusted q<0.05 are included in the table. Pink highlight=increased in obese or overweight vs. healthy-weight (p-Holm<0.05). Green highlight=decreased in obese or overweight vs. healthy-weight (p-Holm<0.05).

Taxon ID	Taxonomy	Level	Normalized Mean	Maximum Cook's Distance	LRT pvalue	LRT qvalue	Fold Change (Obese)	Lower 95% CI (Obese)	Upper 95% CI (Obese)	pvalue (Obese)	p-Holm (Obese)	Fold Change (Over-weight)	Lower 95% CI (Over-weight)	Upper 95% CI (Over-weight)	pvalue (Over-weight)	p-Holm (Over-weight)
787709	Actinobacteria; Actinobacteria; Actinomycetales	Order	3.29	2.95	0.0017	0.01197	1.6	1.22	2.1	0.00066	0.00132	1.04	0.81	1.33	0.74761	0.747608
787709	Actinobacteria; Actinobacteria; Actinomycetales; Actinomycetaceae	Family	2.45	0.34	0.00239	0.01497	1.59	1.22	2.07	0.0005	0.00101	1.19	0.93	1.51	0.15876	0.158758
338754	Bacteroidetes; Bacteroidia; Bacteroidales; Bacteroidaceae; Bacteroides	OTU	8.03	0.63	0.00015	0.00951	0.89	0.58	1.38	0.60271	0.60271	2.19	1.48	3.23	8.11E-05	0.000162
535375	Bacteroidetes; Bacteroidia; Bacteroidales; Bacteroidaceae; Bacteroides; ovatus	Species	66.49	0.08	8.33E-05	0.00176	1.65	1.23	2.21	0.00075	0.00149	0.93	0.72	1.2	0.56505	0.565048
535375	Bacteroidetes; Bacteroidia; Bacteroidales; Bacteroidaceae; Bacteroides; ovatus	OTU	57.89	0.05	0.00036	0.01469	1.52	1.14	2.03	0.00462	0.00924	0.88	0.68	1.14	0.32446	0.32446
276149	Bacteroidetes; Bacteroidia; Bacteroidales; Porphyromonadaceae; Parabacteroides	OTU	11.21	0.04	0.00029	0.01388	0.31	0.18	0.55	5.40E-05	0.00011	0.51	0.31	0.86	0.01107	0.011071
357046	Bacteroidetes; Bacteroidia; Bacteroidales; Rikenellaceae	Family	268.72	0.03	0.01228	0.04724	0.67	0.52	0.87	0.00279	0.00558	0.92	0.73	1.15	0.46113	0.461128
357046	Bacteroidetes; Bacteroidia; Bacteroidales; Rikenellaceae	Genus	247.25	0.23	0.0007	0.0075	0.6	0.46	0.78	0.00011	0.00023	0.88	0.7	1.11	0.28585	0.285851
357046	Bacteroidetes; Bacteroidia; Bacteroidales; Rikenellaceae	Species	240.41	0.22	0.00017	0.00292	0.57	0.43	0.74	2.19E-05	4.38E-05	0.86	0.68	1.09	0.21635	0.216348
357046	Bacteroidetes; Bacteroidia; Bacteroidales; Rikenellaceae	OTU	111.5	0.06	0.00099	0.02648	0.46	0.32	0.68	0.0001	0.0002	0.74	0.52	1.05	0.08822	0.088224
579608	Firmicutes; Bacilli	Class	153.6	0.09	1.04E-12	1.98E-11	2.93	2.19	3.92	6.03E-13	1.21E-12	1.55	1.2	2.01	0.00085	0.000847
1074210	Firmicutes; Bacilli; Gemellales	Order	0.24	0.05	0.00248	0.01197	2.35	1.44	3.81	0.00058	0.00115	1.47	0.92	2.34	0.10332	0.103316
1074210	Firmicutes; Bacilli; Gemellales; Gemellaceae	Family	0.26	0.04	0.00516	0.02582	2.3	1.39	3.82	0.00124	0.00248	1.46	0.9	2.36	0.12266	0.122656
1074210	Firmicutes; Bacilli; Gemellales; Gemellaceae	Genus	0.24	0.05	0.00312	0.02755	2.28	1.41	3.69	0.0008	0.0016	1.5	0.95	2.38	0.08287	0.082867
579608	Firmicutes; Bacilli; Lactobacillales	Order	136	0.04	6.12E-13	1.47E-11	2.87	2.16	3.81	3.26E-13	6.51E-13	1.68	1.3	2.15	5.55E-05	5.55E-05
1107027	Firmicutes; Bacilli; Lactobacillales; Lactobacillaceae	Family	14.78	1.11	3.29E-07	8.23E-06	6.23	3.44	11.29	1.65E-09	3.30E-09	2.49	1.46	4.24	0.0008	0.000796
1107027	Firmicutes; Bacilli; Lactobacillales; Lactobacillaceae; Lactobacillus	Genus	13.84	0.92	9.25E-07	4.48E-05	5.17	2.94	9.09	1.19E-08	2.39E-08	2.4	1.44	4.02	0.00081	0.000806
1107027	Firmicutes; Bacilli; Lactobacillales; Lactobacillaceae; Lactobacillus	Species	12.19	1.18	9.71E-06	0.00031	5.04	2.75	9.24	1.64E-07	3.29E-07	2.36	1.35	4.1	0.00243	0.002428
579608	Firmicutes; Bacilli; Lactobacillales; Streptococcaceae	Family	128.64	0.01	9.34E-09	4.67E-07	2.42	1.8	3.25	4.40E-09	8.80E-09	1.61	1.24	2.09	0.00031	0.000315
579608	Firmicutes; Bacilli; Lactobacillales; Streptococcaceae; Streptococcus	Genus	115.53	0.01	9.17E-11	8.90E-09	2.72	2.02	3.67	4.23E-11	8.46E-11	1.69	1.3	2.2	9.56E-05	9.56E-05
579608	Firmicutes; Bacilli; Lactobacillales; Streptococcaceae; Streptococcus	Species	111.54	0.02	7.18E-10	9.12E-08	2.61	1.94	3.52	2.97E-10	5.94E-10	1.61	1.24	2.1	0.00039	0.00039
4424239	Firmicutes; Bacilli; Lactobacillales; Streptococcaceae; Streptococcus	OTU	0.28	0.04	0.00101	0.02648	2.51	1.53	4.1	0.00025	0.00049	1.74	1.09	2.78	0.0208	0.020796
579608	Firmicutes; Bacilli; Lactobacillales; Streptococcaceae; Streptococcus	OTU	97.39	0.02	4.23E-08	1.91E-05	2.49	1.82	3.41	1.29E-08	2.58E-08	1.43	1.08	1.9	0.0116	0.0116
888300	Firmicutes; Bacilli; Lactobacillales; Streptococcaceae; Streptococcus	OTU	2.81	0.01	8.18E-05	0.00602	2.28	1.57	3.3	1.51E-05	3.02E-05	1.47	1.05	2.07	0.02475	0.024748
967427	Firmicutes; Bacilli; Lactobacillales; Streptococcaceae; Streptococcus	OTU	0.54	0.03	0.00099	0.02648	2.3	1.48	3.56	0.00021	0.00042	1.47	0.97	2.23	0.06712	0.067115
968954	Firmicutes; Bacilli; Lactobacillales; Streptococcaceae; Streptococcus	OTU	13.72	0.03	2.61E-06	0.00047	2.33	1.66	3.26	8.67E-07	1.73E-06	1.55	1.14	2.09	0.00464	0.004641
470382	Firmicutes; Clostridia; Clostridiales	Species	1016.13	0.01	0.00359	0.03508	0.84	0.76	0.93	0.00099	0.00198	0.9	0.82	0.99	0.02853	0.028526
157054	Firmicutes; Clostridia; Clostridiales	OTU	0.17	0.02	0.0009	0.02648	0.48	0.29	0.78	0.00319	0.00319	0.49	0.31	0.76	0.00154	0.003086
178965	Firmicutes; Clostridia; Clostridiales	OTU	1.42	0.01	0.00101	0.02648	0.46	0.31	0.7	0.0002	0.00041	0.87	0.61	1.25	0.44237	0.442374
186918	Firmicutes; Clostridia; Clostridiales	OTU	0.26	0.01	0.00098	0.02648	0.4	0.24	0.67	0.00046	0.00092	0.88	0.56	1.38	0.57338	0.57338
188749	Firmicutes; Clostridia; Clostridiales	OTU	0.71	0	8.32E-05	0.00602	0.56	0.39	0.79	0.00117	0.00234	1.18	0.88	1.59	0.27834	0.278338
191421	Firmicutes; Clostridia; Clostridiales	OTU	0.2	0.21	0.00045	0.017	0.31	0.18	0.54	3.84E-05	7.67E-05	0.56	0.34	0.92	0.02128	0.02128
195946	Firmicutes; Clostridia; Clostridiales	OTU	3.23	0.57	0.00059	0.01977	0.61	0.37	0.99	0.04518	0.04518	0.39	0.25	0.61	3.26E-05	6.52E-05
333114	Firmicutes; Clostridia; Clostridiales	OTU	1.63	0.05	0.00026	0.01332	2.36	1.44	3.85	0.00061	0.00061	2.41	1.53	3.79	0.00015	0.000308
350666	Firmicutes; Clostridia; Clostridiales	OTU	5.57	0.5	0.00016	0.00966	0.25	0.12	0.5	0.00011	0.00022	0.71	0.36	1.42	0.33604	0.33604
358944	Firmicutes; Clostridia; Clostridiales	OTU	3.74	0.31	0.00171	0.03925	1.87	1.32	2.66	0.0004	0.00081	1.47	1.07	2.02	0.01885	0.018849
360890	Firmicutes; Clostridia; Clostridiales	OTU	9.37	0.25	0.0008	0.02494	0.33	0.2	0.54	1.81E-05	3.63E-05	0.62	0.39	0.99	0.04395	0.043954
368025	Firmicutes; Clostridia; Clostridiales	OTU	4.3	0.37	0.00054	0.01946	0.41	0.26	0.64	9.40E-05	0.00019	0.51	0.34	0.77	0.00125	0.001247
369822	Firmicutes; Clostridia; Clostridiales	OTU	3.03	0	0.00017	0.00966	0.54	0.4	0.72	2.34E-05	4.69E-05	0.81	0.63	1.05	0.11167	0.111669
369994	Firmicutes; Clostridia; Clostridiales	OTU	0.79	0.14	8.60E-06	0.00111	0.18	0.09	0.35	2.46E-07	4.91E-07	0.44	0.24	0.81	0.00803	0.008035
509416	Firmicutes; Clostridia; Clostridiales	OTU	8.63	0.02	0.00029	0.01388	0.65	0.52	0.8	7.85E-05	0.00016	0.92	0.76	1.11	0.39467	0.394672
564806	Firmicutes; Clostridia; Clostridiales	OTU	15	0.77	0.00071	0.02244	1.2	0.84	1.71	0.31155	0.31155	0.64	0.47	0.88	0.00604	0.012076
566434	Firmicutes; Clostridia; Clostridiales	OTU	2.95	0.06	0.00056	0.01946	0.35	0.21	0.59	7.78E-05	0.00016	0.52	0.32	0.84	0.00796	0.007956
584765	Firmicutes; Clostridia; Clostridiales	OTU	1.1	2.56	0.00014	0.00917	0.76	0.46	1.26	0.29092	0.29092	2.5	1.59	3.94	6.80E-05	0.000136
649107	Firmicutes; Clostridia; Clostridiales	OTU	1.27	0.08	4.56E-06	0.00069	0.39	0.27	0.56	3.55E-07	7.09E-07	0.71	0.52	0.98	0.03882	0.038821
644244	Firmicutes; Clostridia; Clostridiales; Christensenellaceae	Family	65.38	0.03	0.0024	0.01497	0.57	0.4	0.82	0.00272	0.00544	0.63	0.46	0.87	0.00556	0.005565
644244	Firmicutes; Clostridia; Clostridiales; Christensenellaceae	Genus	69.95	0.01	0.00226	0.02189	0.58	0.4	0.84	0.00377	0.00754	0.62	0.44	0.86	0.0044	0.007538
644244	Firmicutes; Clostridia; Clostridiales; Christensenellaceae	Species	72.26	0.02	0.00275	0.02908	0.58	0.4	0.85	0.00469	0.00931	0.62	0.44	0.86	0.00465	0.00931
234447	Firmicutes; Clostridia; Clostridiales; Christensenellaceae	OTU	0.44	0.78	6.00E-05	0.00543	0.22	0.11	0.45	2.37E-05	4.74E-05	0.35	0.18	0.69	0.00232	0.002317
4410097	Firmicutes; Clostridia; Clostridiales; Christensenellaceae	OTU	1.33	0.04	5.36E-07	0.00012	0.15	0.08	0.3	7.83E-08	1.57E-07	0.31	0.16	0.59	0.00038	0.000382
536584	Firmicutes; Clostridia; Clostridiales; Christensenellaceae	OTU	3.58	0.07	3.39E-07	8.75E-05	0.15	0.08	0.3	6.25E-08	1.25E-07	0.31	0.16	0.59	0.00037	0.000375
555945	Firmicutes; Clostridia; Clostridiales; Clostridiaceae	Family	182.43	0.02	5.34E-07	8.91E-06	0.58	0.48	0.71	1.82E-07	3.64E-07	0.73	0.61	0.87	0.00043	0.000432

Taxon ID	Taxonomy	Level	Normalized Mean	Maximum Cook's Distance	LRT pvalue	LRT qvalue	Fold Change (Obese)	Lower 95% CI (Obese)	Upper 95% CI (Obese)	pvalue (Obese)	p-Holm (Obese)	Fold Change (Over-weight)	Lower 95% CI (Over-weight)	Upper 95% CI (Over-weight)	pvalue (Over-weight)	p-Holm (Over-weight)
780650	Firmicutes; Clostridia; Clostridiales; Clostridiaceae	Genus	23.39	0.06	0.0002	0.00247	0.6	0.47	0.76	3.06E-05	6.11E-05	0.86	0.7	1.06	0.16063	0.160631
780650	Firmicutes; Clostridia; Clostridiales; Clostridiaceae	Species	23.45	0.04	0.00023	0.00293	0.6	0.47	0.76	3.38E-05	6.76E-05	0.86	0.69	1.06	0.15918	0.159177
780650	Firmicutes; Clostridia; Clostridiales; Clostridiaceae	OTU	8.71	0.05	6.89E-05	0.00565	0.28	0.16	0.48	3.86E-06	7.73E-06	0.78	0.47	1.28	0.32367	0.323668
199388	Firmicutes; Clostridia; Clostridiales; Clostridiaceae; Clostridium	OTU	0.22	0	0.00179	0.03968	2.17	1.42	3.32	0.00033	0.00067	1.57	1.04	2.37	0.03062	0.030615
235424	Firmicutes; Clostridia; Clostridiales; Clostridiaceae; Clostridium	OTU	0.29	0.03	0.00034	0.01465	0.31	0.16	0.57	0.00023	0.00046	0.84	0.47	1.5	0.55619	0.556193
555945	Firmicutes; Clostridia; Clostridiales; Clostridiaceae; SMB53	Genus	78.92	0.02	0.00017	0.00231	0.49	0.35	0.68	3.36E-05	6.71E-05	0.67	0.5	0.91	0.00956	0.009557
555945	Firmicutes; Clostridia; Clostridiales; Clostridiaceae; SMB53	Species	81.32	0.03	0.00021	0.00292	0.49	0.34	0.69	4.36E-05	8.72E-05	0.66	0.49	0.9	0.00864	0.008636
316496	Firmicutes; Clostridia; Clostridiales; Clostridiaceae; SMB53	OTU	1.57	0.01	0.00153	0.03596	0.46	0.3	0.71	0.00043	0.00085	0.65	0.44	0.96	0.02997	0.029968
555945	Firmicutes; Clostridia; Clostridiales; Clostridiaceae; SMB53	OTU	83.41	0.04	0.00093	0.02648	0.49	0.34	0.71	0.00015	0.00031	0.7	0.5	0.97	0.03435	0.034354
237991	Firmicutes; Clostridia; Clostridiales; Dehalobacteriaceae	Family	0.66	0.08	2.76E-06	3.46E-05	0.34	0.22	0.53	8.42E-07	1.68E-06	0.6	0.42	0.88	0.008	0.007999
237991	Firmicutes; Clostridia; Clostridiales; Dehalobacteriaceae; Dehalobacterium	Genus	0.68	0.17	1.04E-05	0.00025	0.36	0.23	0.55	2.82E-06	5.63E-06	0.62	0.42	0.9	0.01225	0.012251
237991	Firmicutes; Clostridia; Clostridiales; Dehalobacteriaceae; Dehalobacterium	Species	0.71	0.24	6.37E-06	0.00029	0.34	0.22	0.53	1.50E-06	2.99E-06	0.61	0.41	0.89	0.01078	0.010775
237991	Firmicutes; Clostridia; Clostridiales; Dehalobacteriaceae; Dehalobacterium	OTU	0.5	0.44	2.20E-08	1.91E-05	0.23	0.14	0.39	4.19E-08	8.37E-08	0.38	0.23	0.6	5.60E-05	5.60E-05
1110135	Firmicutes; Clostridia; Clostridiales; Lachnospiraceae	OTU	0.68	1.2	2.43E-05	0.00258	0.38	0.25	0.6	2.63E-05	5.25E-05	0.46	0.31	0.69	0.00019	0.000192
174862	Firmicutes; Clostridia; Clostridiales; Lachnospiraceae	OTU	2.81	0.01	0.00133	0.03197	0.68	0.54	0.86	0.00099	0.00198	0.98	0.8	1.2	0.86884	0.868839
190502	Firmicutes; Clostridia; Clostridiales; Lachnospiraceae	OTU	0.87	0.01	0.00015	0.00961	2.39	1.57	3.63	4.68E-05	9.35E-05	1.84	1.25	2.72	0.00219	0.002185
198362	Firmicutes; Clostridia; Clostridiales; Lachnospiraceae	OTU	0.53	0.02	0.00114	0.02893	1.81	1.32	2.48	0.0002	0.00041	1.37	1.01	1.85	0.04025	0.040245
2017729	Firmicutes; Clostridia; Clostridiales; Lachnospiraceae	OTU	0.75	0.02	0.00209	0.04383	1.78	1.29	2.45	0.0004	0.0008	1.46	1.08	1.97	0.01504	0.015037
305318	Firmicutes; Clostridia; Clostridiales; Lachnospiraceae	OTU	4.41	0.01	9.43E-05	0.00655	0.47	0.34	0.66	8.54E-06	1.71E-05	0.77	0.57	1.03	0.08054	0.080545
310490	Firmicutes; Clostridia; Clostridiales; Lachnospiraceae	OTU	0.25	0.17	6.01E-05	0.00543	1.29	0.82	2.02	0.26863	0.26863	2.3	1.54	3.45	5.13E-05	0.000103
358483	Firmicutes; Clostridia; Clostridiales; Lachnospiraceae	OTU	2.54	0	0.00063	0.02069	0.42	0.28	0.65	8.72E-05	0.00017	0.76	0.52	1.11	0.1533	0.153301
517583	Firmicutes; Clostridia; Clostridiales; Lachnospiraceae	OTU	0.39	0.13	0.00038	0.01517	1.45	1	2.1	0.05294	0.05294	1.98	1.41	2.79	9.03E-05	0.000181
525142	Firmicutes; Clostridia; Clostridiales; Lachnospiraceae	OTU	0.86	0.08	0.00056	0.01946	1.93	1.34	2.76	0.00038	0.00076	1.13	0.81	1.59	0.46869	0.468688
529905	Firmicutes; Clostridia; Clostridiales; Lachnospiraceae	OTU	3.54	0.03	0.00108	0.02786	1.43	1.17	1.74	0.0004	0.00079	1.31	1.09	1.57	0.00357	0.003573
591118	Firmicutes; Clostridia; Clostridiales; Lachnospiraceae	OTU	0.53	0.22	0.00067	0.0215	0.27	0.13	0.54	0.00021	0.00042	0.7	0.36	1.38	0.30302	0.303016
563086	Firmicutes; Clostridia; Clostridiales; Lachnospiraceae; [Ruminococcus]	OTU	1.4	0.02	0.00036	0.01469	0.31	0.19	0.51	4.99E-06	9.99E-06	0.76	0.49	1.19	0.23291	0.232908
1078587	Firmicutes; Clostridia; Clostridiales; Lachnospiraceae; Blautia	Genus	796.25	0.06	0.00015	0.00231	1.38	1.18	1.6	3.35E-05	6.71E-05	1.12	0.98	1.28	0.0869	0.086902
1078587	Firmicutes; Clostridia; Clostridiales; Lachnospiraceae; Blautia	Species	693.77	0.03	0.00118	0.01362	1.3	1.13	1.51	0.00029	0.00057	1.1	0.97	1.24	0.1589	0.158905
175509	Firmicutes; Clostridia; Clostridiales; Lachnospiraceae; Blautia	OTU	1.03	0.01	0.00247	0.04999	1.58	1.18	2.1	0.0019	0.00379	1.53	1.17	2	0.00189	0.003788
187945	Firmicutes; Clostridia; Clostridiales; Lachnospiraceae; Blautia	OTU	2.7	0.14	0.00174	0.03925	1.76	1.06	2.95	0.03022	0.03022	2.41	1.51	3.86	0.00023	0.00045
362037	Firmicutes; Clostridia; Clostridiales; Lachnospiraceae; Blautia	OTU	0.71	1.01	8.34E-05	0.00602	2.94	1.66	5.21	0.00022	0.00044	0.97	0.56	1.66	0.89805	0.89805
366727	Firmicutes; Clostridia; Clostridiales; Lachnospiraceae; Blautia	OTU	0.55	0.12	0.0013	0.03162	2.11	1.36	3.28	0.00092	0.00184	1.97	1.3	2.98	0.00137	0.001844
525378	Firmicutes; Clostridia; Clostridiales; Lachnospiraceae; Blautia	OTU	4.93	0.05	0.00028	0.01388	1.45	1.19	1.75	0.00017	0.00034	1.32	1.11	1.57	0.0016	0.001602
549635	Firmicutes; Clostridia; Clostridiales; Lachnospiraceae; Blautia	OTU	9.64	1.13	7.52E-09	1.36E-05	4.15	2.65	6.49	4.42E-10	8.84E-10	1.48	0.98	2.22	0.06	0.060002
583089	Firmicutes; Clostridia; Clostridiales; Lachnospiraceae; Blautia	OTU	29	0.09	7.94E-06	0.0011	2.18	1.36	3.5	0.00125	0.00125	2.95	1.92	4.53	7.28E-07	1.46E-06
178238	Firmicutes; Clostridia; Clostridiales; Lachnospiraceae; Coprococcus	OTU	18.08	0.01	0.00095	0.02648	0.72	0.58	0.89	0.0022	0.00439	1.05	0.87	1.27	0.6194	0.619397
184770	Firmicutes; Clostridia; Clostridiales; Lachnospiraceae; Coprococcus	OTU	0.81	0.51	0.00249	0.04999	1.85	1.26	2.72	0.00175	0.0035	1.09	0.75	1.57	0.65203	0.652032
187569	Firmicutes; Clostridia; Clostridiales; Lachnospiraceae; Coprococcus	OTU	0.37	0.02	0.00045	0.017	0.61	0.43	0.87	0.00634	0.01269	1.18	0.88	1.59	0.2733	0.2733
188625	Firmicutes; Clostridia; Clostridiales; Lachnospiraceae; Coprococcus	OTU	1.57	0.03	0.00121	0.02994	0.72	0.55	0.95	0.01797	0.03594	1.16	0.92	1.47	0.21176	0.211764
842596	Firmicutes; Clostridia; Clostridiales; Lachnospiraceae; Coprococcus	OTU	5.65	0.07	0.00039	0.01533	0.55	0.41	0.73	5.29E-05	0.00011	0.84	0.65	1.09	0.18222	0.182223
166637	Firmicutes; Clostridia; Clostridiales; Lachnospiraceae; Dorea	OTU	0.7	0.01	0.00101	0.02648	0.28	0.14	0.54	0.00019	0.00037	0.86	0.45	1.63	0.64586	0.645862
584463	Firmicutes; Clostridia; Clostridiales; Lachnospiraceae; Lachnobacterium	Genus	24.45	0.36	3.22E-06	0.0001	1.62	0.99	2.65	0.05332	0.05332	3.09	1.99	4.8	4.70E-07	9.41E-07
584463	Firmicutes; Clostridia; Clostridiales; Lachnospiraceae; Lachnobacterium	Species	23.96	0.29	6.90E-06	0.00029	1.64	1	2.67	0.04855	0.04855	3.01	1.94	4.66	8.84E-07	1.77E-06
584463	Firmicutes; Clostridia; Clostridiales; Lachnospiraceae; Lachnobacterium	OTU	24.98	0.22	0.0002	0.01076	1.62	1.01	2.6	0.04493	0.04493	2.48	1.62	3.81	3.03E-05	6.07E-05
16030	Firmicutes; Clostridia; Clostridiales; Ruminococcaceae	OTU	0.17	0	0.00201	0.04316	0.32	0.16	0.61	0.00065	0.0013	0.65	0.35	1.21	0.1731	0.1731
178015	Firmicutes; Clostridia; Clostridiales; Ruminococcaceae	OTU	0.65	0.01	0.00138	0.03274	1.55	1.07	2.25	0.02005	0.02005	1.91	1.36	2.69	0.00021	0.000415
187489	Firmicutes; Clostridia; Clostridiales; Ruminococcaceae	OTU	0.26	0.07	0.00118	0.02965	0.65	0.42	1	0.04979	0.09958	1.37	0.97	1.94	0.07616	0.09958
187883	Firmicutes; Clostridia; Clostridiales; Ruminococcaceae	OTU	0.67	0.14	0.00058	0.01977	0.78	0.55	1.11	0.16905	0.16905	1.46	1.07	1.98	0.01542	0.030846
190649	Firmicutes; Clostridia; Clostridiales; Ruminococcaceae	OTU	0.53	0.38	3.98E-08	1.91E-05	0.23	0.13	0.38	3.01E-08	6.03E-08	0.4	0.25	0.64	0.00013	0.000129
191555	Firmicutes; Clostridia; Clostridiales; Ruminococcaceae	OTU	1.79	0.01	0.0018	0.03968	1.62	1.22	2.14	0.00074	0.00148	1.13	0.87	1.46	0.36742	0.367415
194654	Firmicutes; Clostridia; Clostridiales; Ruminococcaceae	OTU	1.68	0.03	0.00018	0.0102	0.56	0.42	0.74	3.67E-05	7.34E-05	0.85	0.66	1.09	0.19434	0.194345
195406	Firmicutes; Clostridia; Clostridiales; Ruminococcaceae	OTU	0.4	0.02	4.24E-06	0.00069	0.21	0.12	0.4	8.99E-07	1.80E-06	0.84	0.48	1.46	0.52724	0.527244
230479	Firmicutes; Clostridia; Clostridiales; Ruminococcaceae	OTU	0.45	0.04	0.00244	0.04999	0.32	0.17	0.6	0.00038	0.00076	0.49	0.27	0.89	0.01816	0.018159
293085	Firmicutes; Clostridia; Clostridiales; Ruminococcaceae	OTU	0.22	0.09	0.00094	0.02648	0.34	0.18	0.64	0.00082	0.00164	0.5	0.28	0.9	0.02081	0.020806
311499	Firmicutes; Clostridia; Clostridiales; Ruminococcaceae	OTU	0.58	1.27	3.28E-05	0.00328	2.13	1.48	3.09	5.58E-05	0.00011	1.05	0.73	1.5	0.80928	0.809284

Online Supporting Material

Taxon ID	Taxonomy	Level	Normalized Mean	Maximum Cook's Distance	LRT pvalue	LRT qvalue	Fold Change (Obese)	Lower 95% CI (Obese)	Upper 95% CI (Obese)	pvalue (Obese)	p-Holm (Obese)	Fold Change (Over-weight)	Lower 95% CI (Over-weight)	Upper 95% CI (Over-weight)	pvalue (Over-weight)	p-Holm (Over-weight)
317135	Firmicutes; Clostridia; Clostridiales; Ruminococcaceae	OTU	0.22	0.02	0.00032	0.01424	0.54	0.35	0.84	0.00565	0.0113	1.22	0.84	1.76	0.30162	0.30162
335670	Firmicutes; Clostridia; Clostridiales; Ruminococcaceae	OTU	0.52	0.1	0.00082	0.02504	0.48	0.32	0.71	0.00026	0.00052	0.68	0.48	0.96	0.02853	0.028532
352014	Firmicutes; Clostridia; Clostridiales; Ruminococcaceae	OTU	0.79	0.12	0.00031	0.01424	0.25	0.13	0.47	1.65E-05	3.30E-05	0.6	0.33	1.07	0.0814	0.081398
360761	Firmicutes; Clostridia; Clostridiales; Ruminococcaceae	OTU	8	0.1	6.97E-08	2.47E-05	0.14	0.08	0.25	6.15E-11	1.23E-10	0.63	0.36	1.08	0.0953	0.0953
369827	Firmicutes; Clostridia; Clostridiales; Ruminococcaceae	OTU	3.92	0.17	0.00054	0.01946	0.29	0.16	0.52	3.15E-05	6.29E-05	0.62	0.36	1.06	0.07785	0.077849
4356062	Firmicutes; Clostridia; Clostridiales; Ruminococcaceae	OTU	0.31	0.1	0.00174	0.03925	2.02	1.26	3.26	0.00379	0.00757	0.97	0.61	1.54	0.89752	0.897523
720944	Firmicutes; Clostridia; Clostridiales; Ruminococcaceae	OTU	3.77	0.02	1.05E-05	0.00127	0.48	0.35	0.67	7.94E-06	1.59E-05	0.98	0.74	1.29	0.86847	0.868473
191660	Firmicutes; Clostridia; Clostridiales; Ruminococcaceae; Faecalibacterium; prausnitzii	OTU	0.75	0.49	0.00032	0.01428	0.86	0.61	1.22	0.40922	0.40922	1.58	1.16	2.14	0.00357	0.007138
520774	Firmicutes; Clostridia; Clostridiales; Ruminococcaceae; Faecalibacterium; prausnitzii	OTU	0.36	0.02	0.00218	0.04525	0.58	0.39	0.86	0.00703	0.01406	1.12	0.83	1.51	0.47203	0.472025
188316	Firmicutes; Clostridia; Clostridiales; Ruminococcaceae; Oscillospira	OTU	4.88	0.03	0.00188	0.04099	0.57	0.42	0.78	0.00033	0.00067	0.83	0.64	1.09	0.18645	0.186449
297111	Firmicutes; Clostridia; Clostridiales; Ruminococcaceae; Oscillospira	OTU	1.78	0	8.19E-08	2.47E-05	0.31	0.2	0.48	1.40E-07	2.79E-07	0.4	0.27	0.59	4.74E-06	4.74E-06
346686	Firmicutes; Clostridia; Clostridiales; Ruminococcaceae; Oscillospira	OTU	0.7	0.32	1.46E-06	0.00029	0.22	0.12	0.42	3.16E-06	6.33E-06	0.27	0.15	0.49	1.80E-05	1.80E-05
2979308	Firmicutes; Clostridia; Clostridiales; Ruminococcaceae; Ruminococcus	OTU	9.77	0.3	6.69E-05	0.00565	1.94	1.42	2.63	2.47E-05	4.93E-05	1.62	1.23	2.14	0.00067	0.000674
403701	Firmicutes; Clostridia; Clostridiales; Veillonellaceae	Family	114.26	0.43	8.35E-05	0.0007	1.46	1.17	1.83	0.00099	0.00197	0.93	0.77	1.14	0.50463	0.504629
836693	Firmicutes; Clostridia; SHA-98	Family	0.39	0.02	0.01032	0.04301	0.49	0.31	0.78	0.00256	0.00511	0.8	0.53	1.19	0.26579	0.265789
364722	Firmicutes; Erysipelotrichi; Erysipelotrichales; Erysipelotrichaceae	OTU	0.66	0.02	0.00207	0.04383	0.28	0.15	0.53	0.00011	0.00022	0.44	0.24	0.81	0.00785	0.007847
42372	Firmicutes; Erysipelotrichi; Erysipelotrichales; Erysipelotrichaceae; [Eubacterium]; dolichum	Species	8.46	0.32	4.31E-05	0.00109	2.09	1.46	2.97	4.81E-05	9.62E-05	1.07	0.78	1.47	0.67636	0.676364
1111294	Proteobacteria	Phylum	324.83	0.13	8.67E-06	8.67E-05	1.65	1.33	2.05	5.99E-06	1.20E-05	1.06	0.87	1.28	0.56168	0.561675
334459	Proteobacteria; Betaproteobacteria; Burkholderiales	Family	1.55	0.41	0.00852	0.03874	2.21	1.06	4.61	0.03435	0.0687	0.76	0.39	1.49	0.42726	0.427262
841907	Proteobacteria; Deltaproteobacteria; Desulfovibrionales; Desulfovibrionaceae; Bilophila	OTU	0.79	0.44	0.00024	0.01288	3.7	2.05	6.68	1.34E-05	2.68E-05	2.26	1.3	3.91	0.00366	0.003661
1111294	Proteobacteria; Gammaproteobacteria	Class	128.45	1.08	6.19E-07	5.88E-06	2.92	1.9	4.48	1.11E-06	2.22E-06	1.09	0.74	1.6	0.66595	0.665954
1111294	Proteobacteria; Gammaproteobacteria; Enterobacteriales	Order	106.92	1.13	2.89E-06	3.47E-05	3.12	1.87	5.2	1.28E-05	2.56E-05	1.02	0.64	1.61	0.94337	0.943371
1111294	Proteobacteria; Gammaproteobacteria; Enterobacteriales; Enterobacteriaceae	Family	117.45	0.89	5.85E-06	5.85E-05	2.91	1.71	4.95	8.13E-05	0.00016	0.85	0.53	1.37	0.51366	0.513662
1111294	Proteobacteria; Gammaproteobacteria; Enterobacteriales; Enterobacteriaceae	Genus	87.08	2.07	1.79E-05	0.00035	2.71	1.6	4.6	0.00021	0.00042	0.83	0.52	1.34	0.44868	0.448677
1111294	Proteobacteria; Gammaproteobacteria; Enterobacteriales; Enterobacteriaceae	Species	77.05	2.62	0.00021	0.00292	2.39	1.42	4.03	0.00106	0.00211	0.87	0.54	1.4	0.57007	0.570066
1111294	Proteobacteria; Gammaproteobacteria; Enterobacteriales; Enterobacteriaceae	OTU	85.46	1.67	2.37E-05	0.00258	2.58	1.54	4.31	0.00031	0.00063	0.89	0.56	1.42	0.6253	0.625303
865469	Proteobacteria; Gammaproteobacteria; Pasteurellales	Order	7.42	1.3	0.00249	0.01197	0.42	0.25	0.68	0.00046	0.00091	0.88	0.57	1.36	0.57142	0.571425
865469	Proteobacteria; Gammaproteobacteria; Pasteurellales; Pasteurellaceae	Family	7.75	0.75	0.0047	0.02582	0.42	0.25	0.7	0.00078	0.00156	0.82	0.53	1.29	0.39463	0.394626

Online Supporting Material

Supplemental Table 5. Sex-stratified differentially abundant taxa between obese and healthy-weight participants, and overweight and healthy-weight participants, as detected by the DESeq function in the DESeq2 package. Models were adjusted for age, study, and polyp status. All taxa with LRT FDR -adjusted q<0.05 in either men or women are included in the table. "NA" p- and p-Holm-values indicate strong outlier influence (maximum Cook's distance>15). Pink highlight=increased in obese or overweight vs. healthy-weight (p-Holm<0.05). Green highlight=decreased in obese or overweight vs. healthy-weight (p-Holm<0.05).

MEN

Taxon ID	Taxonomy	Level	Normalized Mean	Maximum Cook's Distance	LRT pvalue	LRT qvalue	Fold Change (Obese)	Lower 95% CI (Obese)	Upper 95% CI (Obese)	pvalue (Obese)	p-Holm (Obese)	Fold Change (Over-weight)	Lower 95% CI (Over-weight)	Upper 95% CI (Over-weight)	pvalue (Over-weight)	p-Holm (Over-weight)
1060029	Actinobacteria; Actinobacteria; Actinomycetales; Corynebacteriaceae	Family	0.54	0.69	1.30E-06	3.42E-05	0.28	0.1	0.73	0.00976	0.00976	0.1	0.04	0.24	1.43E-07	2.85E-07
1060029	Actinobacteria; Actinobacteria; Actinomycetales; Corynebacteriaceae; Corynebacterium	Genus	0.58	0.66	1.47E-06	5.46E-05	0.31	0.12	0.78	0.01269	0.01269	0.11	0.05	0.25	1.24E-07	2.49E-07
338754	Bacteroidetes; Bacteroidia; Bacteroidales; Bacteroidaceae; Bacteroides	OTU	9.93	1.95	0.000142	0.04347	1.52	0.8	2.87	0.19868	0.19868	3.64	2.1	6.3	3.88E-06	7.76E-06
585914	Bacteroidetes; Bacteroidia; Bacteroidales; Porphyromonadaceae; Parabacteroides; distason	Species	70.56	0.09	0.000765	0.01929	2.27	1.39	3.73	0.00114	0.00229	1.05	0.7	1.58	0.8206	0.8206
579608	Firmicutes; Bacilli	Class	140.05	0.2	4.77E-05	9.06E-04	2.78	1.8	4.29	4.07E-06	8.14E-06	1.58	1.1	2.26	0.01216	0.01216
579608	Firmicutes; Bacilli; Lactobacillales	Order	118.79	0.05	5.55E-05	6.67E-04	2.66	1.75	4.04	4.66E-06	9.33E-06	1.68	1.19	2.36	0.00329	0.00329
1107027	Firmicutes; Bacilli; Lactobacillales; Lactobacillaceae	Family	18.09	1.23	1.37E-06	3.4E-05	19.52	8.36	45.57	6.43E-12	1.29E-11	3.07	1.49	6.31	0.00228	0.00228
1107027	Firmicutes; Bacilli; Lactobacillales; Lactobacillaceae; Lactobacillus	Genus	16.94	1.07	1.72E-06	5.5E-05	15.89	7.02	35.98	3.3E-11	6.6E-11	3.22	1.6	6.5	0.0011	0.0011
1107027	Firmicutes; Bacilli; Lactobacillales; Lactobacillaceae; Lactobacillus	Species	15.81	1.27	8.53E-06	0.00054	15.96	6.74	37.82	3.1E-10	6.2E-10	3.44	1.62	7.28	0.00127	0.00127
579608	Firmicutes; Bacilli; Lactobacillales; Streptococcaceae	Family	103.16	0.01	0.058935	0.20755	1.63	1.06	2.51	0.02547	0.05095	1.46	1.03	2.08	0.03444	0.05095
579608	Firmicutes; Bacilli; Lactobacillales; Streptococcaceae; Streptococcus	Genus	93.72	0.03	0.043346	0.17158	1.72	1.11	2.65	0.01484	0.02969	1.46	1.02	2.09	0.03682	0.03682
579608	Firmicutes; Bacilli; Lactobacillales; Streptococcaceae; Streptococcus	Species	89.07	0.06	0.058006	0.24626	1.63	1.06	2.52	0.02619	0.05239	1.47	1.03	2.1	0.03413	0.05239
579608	Firmicutes; Bacilli; Lactobacillales; Streptococcaceae; Streptococcus	OTU	73.16	0.4	0.295411	0.77681	1.39	0.89	2.18	0.14744	0.29488	1.31	0.9	1.9	0.16109	0.29488
514940	Firmicutes; Clostridia	Class	8730.65	0.05	0.002774	0.01757	0.89	0.75	1.05	0.15337	0.15337	1.13	0.99	1.29	0.07516	0.15032
505587	Firmicutes; Clostridia; Clostridiales; [Tissierellaceae]	Family	3.67	2.63	0.008357	0.04643	0.42	0.17	1	0.04984	0.04984	0.27	0.13	0.56	4.72E-04	0.00094
505587	Firmicutes; Clostridia; Clostridiales; [Tissierellaceae]; Finegoldia	Genus	1.43	2.78	0.003976	0.03434	0.38	0.14	1.02	0.05425	0.05425	0.19	0.08	0.45	1.73E-04	0.00035
505587	Firmicutes; Clostridia; Clostridiales; [Tissierellaceae]; Finegoldia	Species	1.33	3.11	0.005921	0.06783	0.39	0.15	1.02	0.05571	0.05571	0.21	0.09	0.49	0.00029	0.00058
644244	Firmicutes; Clostridia; Clostridiales; Christensenellaceae	Family	49.56	0.08	0.00036	0.003	0.59	0.34	1.02	0.05763	0.05763	0.42	0.27	0.65	0.00014	0.00028
644244	Firmicutes; Clostridia; Clostridiales; Christensenellaceae	Genus	53.63	0.09	2.37E-04	0.0045	0.59	0.34	1.04	6.62E-02	6.62E-02	0.4	0.25	0.63	8.7E-05	0.00017
644244	Firmicutes; Clostridia; Clostridiales; Christensenellaceae	Species	56.44	0.09	0.000281	0.0101	0.59	0.34	1.04	0.06976	0.06976	0.39	0.25	0.63	9E-05	0.00018
555945	Firmicutes; Clostridia; Clostridiales; Clostridiaceae	Family	188.38	0.23	0.003039	0.02171	0.61	0.45	0.82	0.00103	0.00205	0.75	0.59	0.96	0.02107	0.02107
780650	Firmicutes; Clostridia; Clostridiales; Clostridiaceae	Genus	24.36	0.15	0.00174	0.01971	0.51	0.35	0.74	0.00038	0.00077	0.82	0.61	1.11	0.20055	0.20055
780650	Firmicutes; Clostridia; Clostridiales; Clostridiaceae	Species	24.28	0.09	0.002244	0.0355	0.51	0.35	0.75	0.00051	0.00102	0.82	0.6	1.11	0.20452	0.20452
780650	Firmicutes; Clostridia; Clostridiales; Clostridiaceae	OTU	10.42	0.08	4.48E-05	0.0201	0.17	0.08	0.35	1.6E-06	3.2E-06	0.58	0.31	1.09	0.09037	0.09037
555945	Firmicutes; Clostridia; Clostridiales; Clostridiaceae; SMB53	Genus	83.82	0.11	0.055379	0.19337	0.56	0.33	0.93	0.02549	0.05099	0.68	0.44	1.04	0.07274	0.07274
555945	Firmicutes; Clostridia; Clostridiales; Clostridiaceae; SMB53	Species	85.73	0.13	0.060588	0.24626	0.57	0.34	0.95	0.03135	0.0627	0.67	0.44	1.03	0.06602	0.06602
237991	Firmicutes; Clostridia; Clostridiales; Dehalobacteriaceae	Family	0.49	0.52	4.47E-03	0.02793	0.47	0.24	0.9	0.02328	0.02328	0.43	0.25	0.75	3.13E-03	6.25E-03
237991	Firmicutes; Clostridia; Clostridiales; Dehalobacteriaceae; Dehalobacterium	Genus	0.53	0.72	0.001867	0.01971	0.44	0.23	0.86	0.01548	0.01548	0.39	0.22	0.69	0.00118	0.00236
237991	Firmicutes; Clostridia; Clostridiales; Dehalobacteriaceae; Dehalobacterium	Species	0.58	0.59	0.001267	0.0266	0.41	0.21	0.81	0.0096	0.0096	0.38	0.21	0.67	0.00081	0.00162
237991	Firmicutes; Clostridia; Clostridiales; Dehalobacteriaceae; Dehalobacterium	OTU	0.53	6.09	0.000225	0.05015	0.24	0.11	0.52	0.00034	0.00054	0.27	0.13	0.54	0.00027	0.00054
1111191	Firmicutes; Clostridia; Clostridiales; Lachnospiraceae; [Ruminococcus]	Genus	174.96	0.1	0.000911	0.01443	0.77	0.6	0.99	0.03951	0.03951	0.68	0.56	0.84	0.00026	0.00053
1111191	Firmicutes; Clostridia; Clostridiales; Lachnospiraceae; [Ruminococcus]; gnavus	Species	98.69	0.41	0.038246	0.17848	0.78	0.59	1.04	0.08965	0.08965	0.75	0.59	0.94	0.01336	0.02672
549635	Firmicutes; Clostridia; Clostridiales; Lachnospiraceae; Blautia	OTU	8.8	3.39	3.02E-09	5.4E-06	8.38	4.45	15.78	4.7E-11	9.4E-11	2.1	1.21	3.66	0.00853	0.00853
187782	Firmicutes; Clostridia; Clostridiales; Lachnospiraceae; Coprococcus	OTU	0.54	4.4	1.79E-05	0.01072	1.16	0.57	2.36	0.68988	0.68988	3.81	2.04	7.09	2.5E-05	5E-05
584463	Firmicutes; Clostridia; Clostridiales; Lachnospiraceae; Lachnobacterium	Genus	25.5	1.53	0.003021	0.0287	1.64	0.81	3.3	0.16874	0.16874	2.86	1.59	5.15	0.00047	0.00094
584463	Firmicutes; Clostridia; Clostridiales; Lachnospiraceae; Lachnobacterium	Species	25.27	1.62	0.00388	0.04889	1.57	0.78	3.14	0.20619	0.20619	2.76	1.54	4.97	0.00068	0.00136
4356062	Firmicutes; Clostridia; Clostridiales; Ruminococcaceae	OTU	0.27	0.11	1.45E-04	0.04347	3.2	1.62	6.35	0.00084	0.00169	1.08	0.57	2.05	0.81215	0.81215
403701	Firmicutes; Clostridia; Clostridiales; Veillonellaceae	Family	124.26	1.08	2.68E-04	0.00268	1.65	1.22	2.23	0.00111	0.00223	1.01	0.79	1.29	9.45E-01	9.45E-01
264967	Firmicutes; Clostridia; Clostridiales; Veillonellaceae; Megasphaera	Genus	8.38	0.06	1.33E-03	1.81E-02	1.16	0.35	3.85	0.80261	0.80261	0.18	0.06	0.57	3.78E-03	7.57E-03
264967	Firmicutes; Clostridia; Clostridiales; Veillonellaceae; Megasphaera	Species	7.76	0.07	2.25E-03	3.55E-02	1.17	0.38	3.62	0.78158	0.78158	0.21	0.07	0.65	6.46E-03	1.29E-02
580008	Firmicutes; Erysipelotrichi; Erysipelotrichales; Erysipelotrichaceae	Species	80.69	0.02	3.31E-03	4.64E-02	0.67	0.46	0.97	3.51E-02	7.02E-02	1.19	0.88	1.62	0.25687	0.25687
1111294	Proteobacteria	Phylum	326.84	0.3	2.46E-04	0.00246	1.5	1.1	2.05	1.06E-02	2.13E-02	0.86	0.67	1.12	0.26022	0.26022
334459	Proteobacteria; Betaproteobacteria; Burkholderiales	Family	1.19	0.18	0.000102	0.00128	1.25	0.49	3.16	0.64468	0.64468	0.24	0.11	0.54	0.00055	0.00111
334459	Proteobacteria; Betaproteobacteria; Burkholderiales	Genus	1.25	0.33	0.000122	0.0029	1.32	0.54	3.24	0.53996	0.53996	0.26	0.12	0.58	0.00092	0.00183
334459	Proteobacteria; Betaproteobacteria; Burkholderiales	Species	1.14	0.21	0.00032	0.0101	1.23	0.51	2.94	0.64801	0.64801	0.28	0.13	0.61	0.00131	0.00262
1111294	Proteobacteria; Gammaproteobacteria	Class	151.18	1.31	0.000106	0.00101	2.08	1.11	3.9	0.02228	0.04456	0.64	0.38	1.09	0.09829	0.09829
1111294	Proteobacteria; Gammaproteobacteria; Enterobacteriales	Order	117.29	1.51	1.07E-05	0.00026	1.3	0.62	2.72	0.49427	0.49427	0.31	0.17	0.59	0.00028	0.00056
1111294	Proteobacteria; Gammaproteobacteria; Enterobacteriales; Enterobacteriaceae	Family	116.77	1.03	1.14E-05	0.00019	1.18	0.55	2.51	0.67662	0.67662	0.29	0.15	0.55	0.00013	0.00025
1111294	Proteobacteria; Gammaproteobacteria; Enterobacteriales; Enterobacteriaceae	Genus	74.78	1.53	3.93E-08	3.7E-06	1.51	0.72	3.19	0.27882	0.27882	0.23	0.12	0.42	3.8E-06	7.5E-06
1111294	Proteobacteria; Gammaproteobacteria; Enterobacteriales; Enterobacteriaceae	Species	67.19	1.43	1.95E-07	2.5E-05	1.34	0.64	2.79	0.43311	0.43311	0.24	0.13	0.44	5.5E-06	1.1E-05
1111294	Proteobacteria; Gammaproteobacteria; Enterobacteriales; Enterobacteriaceae	OTU	74.27	0.81	1.53E-07	0.00014	1.4	0.7	2.77	0.33964	0.33964	0.27	0.15	0.48	1.46E-05	2.9E-05
3121406	Synergistetes; Synergistia	Class	0.57	4.64	0.006931	0.03292	0.65	0.25	1.65	0.36367	0.36367	0.27	0.12	0.62	0.00204	0.00407

WOMEN

Taxon ID	Taxonomy	Level	Normalized Mean	Maximum Cook's Distance	LRT pvalue	LRT qvalue	Fold Change (Obese)	Lower 95% CI (Obese)	Upper 95% CI (Obese)	pvalue (Obese)	p-Holm (Obese)	Fold Change (Over-weight)	Lower 95% CI (Over-weight)	Upper 95% CI (Over-weight)	pvalue (Over-weight)	p-Holm (Over-weight)
1060029	Actinobacteria; Actinobacteria; Actinomycetales; Corynebacteriaceae	Family	1.07	19.89	NA	NA	3.98	1.63	9.72	NA	NA	3.14	1.3	7.56	NA	NA
1060029	Actinobacteria; Actinobacteria; Actinomycetales; Corynebacteriaceae; Corynebacterium	Genus	0.93	19.5	NA	NA	4.06	1.67	9.86	NA	NA	3.25	1.35	7.81	NA	NA
338754	Bacteroidetes; Bacteroidia; Bacteroidales; Bacteroidaceae; Bacteroides	OTU	6.06	0.12	0.276	0.66246	0.67	0.39	1.15	0.1465	0.293009	1.05	0.62	1.79	0.8503	0.8503
585914	Bacteroidetes; Bacteroidia; Bacteroidales; Porphyromonadaceae; Parabacteroides; distason	Species	117.88	0.91	0.34326	0.71663	0.71	0.41	1.21	0.20466	0.409313	0.73	0.43	1.23	0.2315	0.40931
579608	Firmicutes; Bacilli	Class	162.34	0.04	5.7E-07	1.1E-05	2.74	1.86	4.02	3.1E-07	6.26E-07	1.52	1.04	2.21	0.0287	0.02866
579608	Firmicutes; Bacilli; Lactobacillales	Order	157.38	0.07	1.2E-07	2.9E-06	2.92	1.98	4.31	7.1E-08	1.42E-07	1.61	1.1	2.35	0.0138	0.01375
1107027	Firmicutes; Bacilli; Lactobacillales; Lactobacillaceae	Family	10.32	186.45	NA	NA	2.02	0.95	4.3	NA	NA	2.31	1.1	4.86	NA	NA
1107027	Firmicutes; Bacilli; Lactobacillales; Lactobacillaceae; Lactobacillus	Genus	9.76	186.27	NA	NA	1.86	0.86	4.02	NA	NA	2.19	1.03	4.66	NA	NA
1107027	Firmicutes; Bacilli; Lactobacillales; Lactobacillaceae; Lactobacillus	Species	7.72	226.54	NA	NA	1.58	0.67	3.74	NA	NA	2.04	0.87	4.75	NA	NA
579608	Firmicutes; Bacilli; Lactobacillales; Streptococcaceae	Family	161.36	0.04	1.99E-08	8.97E-07	3.18	2.13	4.75	1.63E-08	3.26E-08	1.55	1.05	2.3	0.0267	0.02666
579608	Firmicutes; Bacilli; Lactobacillales; Streptococcaceae; Streptococcus	Genus	142.98	0.03	1.2E-09	1.1E-07	3.61	2.39	5.46	1E-09	2E-09	1.67	1.12	2.49	0.0124	0.01239
579608	Firmicutes; Bacilli; Lactobacillales; Streptococcaceae; Streptococcus	Species	140.81	0.03	1.9E-09	2.3E-07	3.6	2.37	5.47	1.93E-09	3.86E-09	1.56	1.04	2.34	0.032	0.03199
579608	Firmicutes; Bacilli; Lactobacillales; Streptococcaceae; Streptococcus	OTU	125.62	0.03	1.4E-08	2.5E-05	3.49	2.26	5.39	1.6E-08	3.16E-08	1.32	0.86	2.01	0.2012	0.20117
514940	Firmicutes; Clostridia	Class	8603.85	0.1	0.95101	0.95101	0.98	0.84	1.15	0.84766	1	1.01	0.87	1.18	0.8705	1
505587	Firmicutes; Clostridia; Clostridiales; [Tissierellaceae]	Family	5.76	5.82	0.00046	0.00416	4.36	2.28	8.36	8.9E-06	1.79E-05	1.88	0.99	3.57	0.0539	0.0539
505587	Firmicutes; Clostridia; Clostridiales; [Tissierellaceae]; Finegoldia	Genus	1.28	15.42	NA	NA	4.81	1.96	11.8	NA	NA	2.66	1.09	6.48	NA	NA
505587	Firmicutes; Clostridia; Clostridiales; [Tissierellaceae]; Finegoldia	Species	1.1	14.05	3.95E-03	0.0336	5.22	2.08	13.13	NA	NA	3.27	1.31	8.16	NA	NA
644244	Firmicutes; Clostridia; Clostridiales; Christensenellaceae	Family	82.83	0.11	0.01273	0.05888	0.49	0.31	0.79	0.00324	0.006477	1.03	0.65	1.61	0.9145	0.91448
644244	Firmicutes; Clostridia; Clostridiales; Christensenellaceae	Genus	88.1	0.04	0.0167	0.08549	0.51	0.31	0.82	0.00596	0.011911	1.06	0.66	1.71	0.7956	0.79563
644244	Firmicutes; Clostridia; Clostridiales; Christensenellaceae	Species	92.56	0.05	0.02252	0.11165	0.5	0.3	0.83	0.007	0.014003	1.05	0.64	1.7	0.8528	0.8528
555945	Firmicutes; Clostridia; Clostridiales; Clostridiaceae	Family	175.63	0.02	8.05E-05	9.06E-04	0.57	0.43	0.74	4.84E-05	9.68E-05	0.66	0.51	0.87	0.0026	0.00261
780650	Firmicutes; Clostridia; Clostridiales; Clostridiaceae	Genus	22.62	0.02	5.53E-02	0.20056	0.68	0.5	0.93	1.52E-02	3.03E-02	0.82	0.61	1.11	0.207	0.207
780650	Firmicutes; Clostridia; Clostridiales; Clostridiaceae	Species	22.58	0.02	5.02E-02	0.19894	0.67	0.49	0.92	1.44E-02	2.88E-02	0.8	0.59	1.09	0.1596	0.15956
780650	Firmicutes; Clostridia; Clostridiales; Clostridiaceae	OTU	6.85	0.69	0.22065	0.61684	0.51	0.23	1.11	9.12E-02	0.182382	0.99	0.46	2.13	0.9833	0.98334
555945	Firmicutes; Clostridia; Clostridiales; Clostridiaceae; SMB53	Genus	76.42	0.01	0.00178	0.02217	0.43	0.27	0.68	0.0003	0.000597	0.62	0.4	0.97	0.0374	0.03742
555945	Firmicutes; Clostridia; Clostridiales; Clostridiaceae; SMB53	Species	77.43	0.01	1.74E-03	0.02043	0.43	0.27	0.68	3.35E-04	6.70E-04	0.6	0.38	0.94	0.0261	0.02608
237991	Firmicutes; Clostridia; Clostridiales; Dehalobacteriaceae	Family	0.87	0.13	4.1E-07	9.2E-06	0.24	0.14	0.41	1.3E-07	2.55E-07	0.9	0.56	1.45	0.6714	0.67143
237991	Firmicutes; Clostridia; Clostridiales; Dehalobacteriaceae; Dehalobacterium	Genus	0.84	0.16	3.5E-06	0.00015	0.27	0.16	0.46	2.1E-06	4.15E-06	1.03	0.63	1.68	0.9143	0.91428
237991	Firmicutes; Clostridia; Clostridiales; Dehalobacteriaceae; Dehalobacterium	Species	0.89	0.25	5.1E-06	0.0003	0.26	0.15	0.46	2.7E-06	5.49E-06	1.05	0.63	1.76	0.8439	0.84392
237991	Firmicutes; Clostridia; Clostridiales; Dehalobacteriaceae; Dehalobacterium	OTU	0.47	0.02	0.00052	0.0285	0.29	0.15	0.54	9.7E-05	0.000193	0.75	0.41	1.35	0.3376	0.33756
1111191	Firmicutes; Clostridia; Clostridiales; Lachnospiraceae; [Ruminococcus]	Genus	199.07	0.2	0.00091	0.01321	1.57	1.23	2.02	0.00038	0.000766	1.38	1.08	1.75	0.0101	0.01015
1111191	Firmicutes; Clostridia; Clostridiales; Lachnospiraceae; [Ruminococcus]; gnavus	Species	104.67	4.8	5.5E-05	0.0013	2.05	1.48	2.83	1.3E-05	2.6E-05	1.53	1.12	2.09	0.0077	0.00775
549635	Firmicutes; Clostridia; Clostridiales; Lachnospiraceae; Blautia	OTU	10.34	0.29	0.23371	0.62783	1.58	0.87	2.88	0.13424	0.268472	1.49	0.83	2.68	0.1831	0.26847
187782	Firmicutes; Clostridia; Clostridiales; Lachnospiraceae; Coprococcus	OTU	0.98	17.29	NA	NA	1.23	0.57	2.63	NA	NA	0.93	0.43	1.99	NA	NA
584463	Firmicutes; Clostridia; Clostridiales; Lachnospiraceae; Lachnobacterium	Genus	23.61	0.21	0.00321	0.03108	1.28	0.64	2.55	0.4884	0.488397	3.02	1.54	5.93	0.0013	0.00261
584463	Firmicutes; Clostridia; Clostridiales; Lachnospiraceae; Lachnobacterium	Species	22.95	0.19	0.00532	0.0422	1.36	0.67	2.74	0.39461	0.394615	3	1.51	5.95	0.0017	0.00342
4356062	Firmicutes; Clostridia; Clostridiales; Ruminococcaceae	OTU	0.34	0.21	7.73E-01	9.63E-01	1.24	0.65	2.34	5.15E-01	1.00E+00	1	0.52	1.91	0.9993	1
403701	Firmicutes; Clostridia; Clostridiales; Veillonellaceae	Family	104.55	1.91	2.12E-02	0.07937	1.36	0.97	1.91	7.41E-02	1.48E-01	0.81	0.58	1.13	0.2105	0.21046
264967	Firmicutes; Clostridia; Clostridiales; Veillonellaceae; Megasphaera	Genus	15.04	277.66	NA	NA	1.15	0.39	3.37	NA	NA	0.81	0.27	2.4	NA	NA
264967	Firmicutes; Clostridia; Clostridiales; Veillonellaceae; Megasphaera	Species	15.08	281.89	NA	NA	1.22	0.36	4.1	NA	NA	0.79	0.23	2.7	NA	NA
580008	Firmicutes; Erysipelotrichi; Erysipelotrichales; Erysipelotrichaceae	Species	83.67	0.26	0.75126	0.91502	0.89	0.66	1.21	0.45596	0.911917	0.98	0.73	1.32	0.8837	0.91192
1111294	Proteobacteria	Phylum	319.48	0.16	0.00399	0.03994	1.65	1.22	2.22	0.00114	0.002272	1.36	1.02	1.83	0.0378	0.0378
334459	Proteobacteria; Betaproteobacteria; Burkholderiales	Family	1.96	1.96	4.05E-02	1.30E-01	3.46	1.33	9.03	1.12E-02	2.24E-02	2.05	0.79	5.33	0.1386	0.13855
334459	Proteobacteria; Betaproteobacteria; Burkholderiales	Genus	1.87	2.03	3.22E-02	1.40E-01	3.56	1.36	9.3	9.57E-03	1.91E-02	2.44	0.94	6.34	0.0668	0.06675
334459	Proteobacteria; Betaproteobacteria; Burkholderiales	Species	1.77	3.11	3.44E-02	1.58E-01	3.59	1.33	9.66	1.15E-02	2.29E-02	2.68	1.01	7.17	0.0487	0.04866
1111294	Proteobacteria; Gammaproteobacteria	Class	98.25	1.88	0.00074	0.00701	3.07	1.76	5.34	7.2E-05	0.000144	1.81	1.05	3.1	0.0323	0.03225
1111294	Proteobacteria; Gammaproteobacteria; Enterobacteriales	Order	99.51	1.74	3.2E-07	3.8E-06	8.46	4.29	16.68	6.93E-10	1.39E-09	3.62	1.86	7.02	0.0001	0.00015
1111294	Proteobacteria; Gammaproteobacteria; Enterobacteriales; Enterobacteriaceae	Family	118.6	2.58	6.9E-06	0.0001	6.8	3.44	13.44	3.5E-08	6.94E-08	2.51	1.29	4.88	0.0069	0.00686
1111294	Proteobacteria; Gammaproteobacteria; Enterobacteriales; Enterobacteriaceae	Genus	101.4	3.61	8.3E-05	0.0018	6.21	3.02	12.78	7E-07	1.41E-06	3.01	1.49	6.11	0.0022	0.00222
1111294	Proteobacteria; Gammaproteobacteria; Enterobacteriales; Enterobacteriaceae	Species	93.36	4.76	8.1E-05	0.0016	6.09	2.93	12.63	1.2E-06	2.48E-06	3.34	1.64	6.82	0.0009	0.00092
1111294	Proteobacteria; Gammaproteobacteria; Enterobacteriales; Enterobacteriaceae	OTU	97.9	3.91	6E-05	0.00704	5.44	2.66	11.11	3.4E-06	6.82E-06	3.29	1.63	6.64	0.0009	0.00087
3121406	Synergistetes; Synergistia	Class	1.17	1.86	7.44E-02	0.23561	0.76	0.35	1.68	5.05E-01	5.05E-01	1.93	0.9	4.17	0.0924	0.18482

Supplemental Table 6. Differentially abundant KEGG pathways between obese and healthy-weight participants, as detected by the DESeq function in the DESeq2 package (no pathways were identified as differentially abundant between overweight and healthy-weight participants). Models were adjusted for age, sex, polyp status, and study. A priori pathways and pathways with an LRT FDR-adjusted q<0.05 are included in the table.

KEGG Pathway	Normalized Mean	Maximum Cook's Distance	LRT pvalue	LRT qvalue	Fold Change (Obese)	Lower 95% CI (Obese)	Upper 95% CI (Obese)	pvalue (Obese)	p-Holm (Obese)	Fold Change (Overweight)	Lower 95% CI (Overweight)	Upper 95% CI (Overweight)	pvalue (Overweight)	p-Holm (Overweight)
**a priori pathways**														
Metabolism; Carbohydrate Metabolism; Butanoate metabolism	99435.25	0	0.16393	0.8378	0.99	0.98	1	0.056533	0.113065	1	0.99	1	0.310963373	0.3109634
Metabolism; Carbohydrate Metabolism; Propanoate metabolism	80024.82	0	0.80465	0.9613	1	0.99	1.01	0.513401	1	1	0.99	1.01	0.908463497	1
Metabolism; Energy Metabolism; Methane metabolism	234027.29	0.01	0.83392	0.9613	1	0.99	1.01	0.863126	1	1	0.99	1.01	0.67574402	1
Metabolism; Glycan Biosynthesis and Metabolism; Lipopolysaccharide biosynthesis	25560.99	0.02	0.56157	0.9173	1	0.99	1	0.327146	0.327146	0.99	0.98	1	0.112329829	0.2246596
Metabolism; Lipid Metabolism; Secondary bile acid biosynthesis	7647.86	0.02	0.26829	0.9051	1.01	1	1.03	0.08298	0.16596	1.01	0.99	1.02	0.389159982	0.38916
**FDR-adjusted significant pathways**														
Metabolism; Lipid Metabolism; alpha-Linolenic acid metabolism	217.71	0.17	8.29E-06	0.0015	1.01	1.01	1.02	8.02E-08	1.60E-07	1	1	1.01	0.241213285	0.2412133

Online Supporting Material

Supplemental Table 7. Study-stratified differentially abundant taxa between obese and healthy-weight participants, and overweight and healthy-weight participants, as detected by the DESeq function in the DESeq2 package. Models were adjusted for age, sex, and polyp status. All taxa with LRT-FDR-adjusted q<0.05 in either the CDC or NYU studies are included in the table. "NA" p- and p-Holm-values indicate strong outlier influence (maximum Cook's distance>15). Pink highlight=increased in obese or overweight vs. healthy-weight (p-Holm<0.05). Green highlight=decreased in obese or overweight vs. healthy-weight (p-Holm<0.05).

			CDC														NYU													
Taxon ID	Taxonomy	Level	Normalized Mean	Maximum Cook's Distance	LRT pvalue	LRT qvalue	Fold Change (Obese)	Lower 95% CI (Obese)	Upper 95% CI (Obese)	pvalue (Obese)	p-Holm (Obese)	Fold Change (Over-weight)	Lower 95% CI (Over-weight)	Upper 95% CI (Over-weight)	pvalue (Over-weight)	p-Holm (Over-weight)	Normalized Mean	Maximum Cook's Distance	LRT pvalue	LRT qvalue	Fold Change (Obese)	Lower 95% CI (Obese)	Upper 95% CI (Obese)	pvalue (Obese)	p-Holm (Obese)	Fold Change (Over-weight)	Lower 95% CI (Over-weight)	Upper 95% CI (Over-weight)	pvalue (Over-weight)	p-Holm (Over-weight)
290455	Bacteroidetes; Bacteroidia; Bacteroidales	Family	14.65	0.16	0.0049	0.03061	4.61	1.39	15.27	0.0123	0.01965	4.6	1.44	14.65	0.00983	0.01965	2.25	10.79	1	1	1.85	0.47	7.26	NA	NA	1.46	0.4	5.37	NA	NA
186866	Bacteroidetes; Bacteroidia; Bacteroidales; Bacteroidaceae; Bacteroides	OTU	1.07	2.86	6.93E-05	0.00623	1.42	0.68	2.97	0.35197	0.35197	5.01	2.45	10.21	9.50E-06	1.90E-05	0.68	29.39	NA	NA	0.38	0.11	1.36	NA	NA	0.29	0.07	1.14	NA	NA
276149	Bacteroidetes; Bacteroidia; Bacteroidales; Porphyromonadaceae; Parabacteroides	OTU	15.84	0.05	0.00088	0.03098	0.28	0.15	0.54	0.00013	0.00025	0.54	0.29	1	0.0508	0.0508	3.88	0.39	0.045824	0.887091	0.31	0.1	0.93	0.03726	0.03726	0.32	0.14	0.77	0.0104	0.0207
357046	Bacteroidetes; Bacteroidia; Bacteroidales; Rikenellaceae	Genus	425.15	0.25	0.00201	0.02434	0.58	0.43	0.78	0.00028	0.00056	0.78	0.59	1.02	0.0739	0.0739	51.84	0.15	0.199794	0.684483	0.61	0.34	1.1	0.10232	0.20465	1.1	0.73	1.65	0.6542	0.6542
357046	Bacteroidetes; Bacteroidia; Bacteroidales; Rikenellaceae	Species	408.17	0.37	0.0007	0.01279	0.55	0.41	0.74	8.35E-05	0.00017	0.76	0.58	1.01	0.05593	0.05593	52.82	0.25	0.158243	0.678183	0.56	0.31	1.01	0.05199	0.10399	1.02	0.67	1.54	0.9308	0.9308
357046	Bacteroidetes; Bacteroidia; Bacteroidales; Rikenellaceae	OTU	167.49	0.04	0.00095	0.03109	0.44	0.29	0.68	0.00016	0.00032	0.56	0.37	0.84	0.00476	0.00476	25.84	0.37	0.248963	1	0.5	0.21	1.21	0.12404	0.24807	1.14	0.59	2.17	0.6993	0.6993
579608	Firmicutes; Bacilli	Class	245.61	0.09	3.06E-07	2.91E-06	2.38	1.73	3.28	8.68E-08	1.74E-07	1.4	1.04	1.9	0.02754	0.02754	46.85	14.38	9.84E-07	1.67E-05	5.13	2.6	10.13	2.46E-06	4.93E-06	2.05	1.26	3.32	0.0036	0.0036
1084865	Firmicutes; Bacilli; Bacillales	Order	0.31	0.02	0.00184	0.01195	2.06	1.07	3.96	0.03078	0.06155	0.68	0.36	1.31	0.2524	0.2524	0.07	0.46	1	1	0.85	0.18	4	0.83521	1	1.01	0.26	3.95	0.9901	1
579608	Firmicutes; Bacilli; Lactobacillales	Order	216.69	0.06	1.73E-07	4.14E-06	2.41	1.77	3.29	2.11E-08	4.23E-08	1.56	1.17	2.09	0.00265	0.00265	46.86	11.71	8.61E-08	1.98E-06	5.81	2.97	11.38	2.81E-07	5.61E-07	1.88	1.16	3.03	0.01	0.01
1107027	Firmicutes; Bacilli; Lactobacillales; Lactobacillaceae	Family	24.17	0.93	5.84E-05	0.00073	5.71	2.92	11.13	3.29E-07	6.58E-07	3.6	1.91	6.79	7.80E-05	7.80E-05	3.72	5.99	0.002548	0.022935	4.23	1.43	12.57	0.0093	0.0186	0.9	0.38	2.13	0.8062	0.8062
1107027	Firmicutes; Bacilli; Lactobacillales; Lactobacillaceae; Lactobacillus	Genus	21.04	0.73	0.00011	0.00269	4.78	2.49	9.16	2.55E-06	5.10E-06	2.93	1.57	5.45	0.00071	0.00071	3.5	5.3	0.002038	0.053612	4.89	1.49	16.04	0.00889	0.01778	0.9	0.36	2.23	0.8169	0.8169
1107027	Firmicutes; Bacilli; Lactobacillales; Lactobacillaceae; Lactobacillus	Species	17.67	1.35	0.0006	0.01275	4.05	2.02	8.13	8.16E-05	0.00016	3.03	1.56	5.9	0.00111	0.00111	2.96	6.04	0.002015	0.048369	4.92	1.58	15.27	0.00583	0.01167	1.02	0.41	2.57	0.9596	0.9596
579608	Firmicutes; Bacilli; Lactobacillales; Streptococcaceae	Family	212.24	0.02	0.0003	0.00298	1.91	1.39	2.63	6.93E-05	0.00014	1.54	1.14	2.09	0.00494	0.00494	36.35	11.16	2.33E-05	0.000525	4.36	2.19	8.68	2.86E-05	5.73E-05	1.5	0.91	2.48	0.1081	0.1081
579608	Firmicutes; Bacilli; Lactobacillales; Streptococcaceae; Streptococcus	Genus	195.47	0.02	2.89E-06	0.00028	2.33	1.68	3.23	4.44E-07	8.87E-07	1.6	1.18	2.19	0.0028	0.0028	34.84	8.3	1.95E-05	0.001755	4.86	2.33	10.11	2.40E-05	4.80E-05	1.58	0.94	2.67	0.0858	0.0858
579608	Firmicutes; Bacilli; Lactobacillales; Streptococcaceae; Streptococcus	Species	182.5	0.01	1.15E-05	0.00074	2.21	1.59	3.06	1.91E-06	3.82E-06	1.54	1.13	2.09	0.00651	0.00651	32.39	9.16	3.68E-05	0.004418	4.37	2.16	8.87	4.31E-05	8.61E-05	1.63	0.98	2.73	0.0614	0.0614
4424239	Firmicutes; Bacilli; Lactobacillales; Streptococcaceae; Streptococcus	OTU	0.43	0.01	0.00161	0.04135	2.63	1.55	4.47	0.00034	0.00068	1.85	1.11	3.11	0.01938	0.01938	0.05	0.24	0.869786	1	1.29	0.35	4.81	0.69966	1	1.26	0.33	4.88	0.7374	1
579608	Firmicutes; Bacilli; Lactobacillales; Streptococcaceae; Streptococcus	OTU	144.5	0.02	9.99E-06	0.00163	2.24	1.6	3.14	2.96E-06	5.92E-06	1.31	0.95	1.81	0.09659	0.09659	27.87	10.94	0.001154	0.230946	3.76	1.72	8.21	0.00088	0.00176	1.47	0.83	2.6	0.1893	0.1893
888300	Firmicutes; Bacilli; Lactobacillales; Streptococcaceae; Streptococcus	OTU	3.81	0.01	0.00017	0.01182	2.35	1.57	3.51	3.31E-05	6.61E-05	1.48	1.01	2.18	0.04515	0.04515	1.26	1.86	0.254942	1	2.16	0.81	5.71	0.12234	0.24468	1.37	0.65	2.89	0.4148	0.4148
967427	Firmicutes; Bacilli; Lactobacillales; Streptococcaceae; Streptococcus	OTU	0.77	0.06	0.00173	0.0439	2.37	1.47	3.81	0.00038	0.00076	1.48	0.93	2.35	0.09837	0.09837	0.18	0.48	0.726454	1	1.53	0.46	5.08	0.48423	0.96847	1.27	0.47	3.4	0.6402	0.9685
968954	Firmicutes; Bacilli; Lactobacillales; Streptococcaceae; Streptococcus	OTU	18.61	0.02	0.00066	0.02814	1.9	1.33	2.7	0.00041	0.00081	1.13	0.8	1.58	0.49092	0.49092	5.96	15.75	NA	NA	4.43	1.82	10.8	NA	NA	2.5	1.28	4.87	NA	NA
178965	Firmicutes; Clostridia; Clostridiales	OTU	2.17	0.01	0.00195	0.04795	0.48	0.31	0.74	0.00096	0.00192	0.96	0.64	1.45	0.84819	0.84819	0.27	0.35	0.67376	1	0.64	0.22	1.83	0.40336	0.80671	0.73	0.33	1.6	0.4261	0.8067
188749	Firmicutes; Clostridia; Clostridiales	OTU	0.92	0.01	9.23E-05	0.00754	0.57	0.39	0.83	0.0036	0.0072	1.27	0.9	1.79	0.17393	0.17393	0.35	0.03	0.644633	1	0.65	0.26	1.62	0.35353	0.70706	0.98	0.52	1.84	0.9391	0.9391
191421	Firmicutes; Clostridia; Clostridiales	OTU	0.33	0.54	0.00078	0.0286	0.27	0.15	0.5	2.69E-05	5.38E-05	0.55	0.32	0.96	0.03415	0.03415	0.02	0.05	0.940053	1	0.89	0.32	2.48	0.82552	1	0.85	0.25	2.94	0.8012	1
195946	Firmicutes; Clostridia; Clostridiales	OTU	5.04	0.7	2.32E-05	0.00321	0.37	0.22	0.62	0.00018	0.00018	0.31	0.19	0.51	4.13E-06	8.25E-06	0.48	4.03	0.024775	0.761483	3.07	0.96	9.81	0.05879	0.11759	0.79	0.3	2.1	0.6314	0.6314
333114	Firmicutes; Clostridia; Clostridiales	OTU	2.44	0.12	0.0009	0.03098	2.44	1.42	4.21	0.0013	0.0013	2.59	1.54	4.36	0.00036	0.00072	0.35	0.66	0.266222	1	2.08	0.68	6.34	0.19725	0.3945	1.8	0.73	4.47	0.2031	0.3945
350666	Firmicutes; Clostridia; Clostridiales	OTU	7.13	0.57	0.00141	0.03826	0.28	0.13	0.61	0.00154	0.00309	0.96	0.44	2.1	0.92125	0.92125	2.91	4.72	0.03399	0.872045	0.27	0.07	1.01	0.05238	0.10475	0.33	0.09	1.23	0.1	0.1048
360890	Firmicutes; Clostridia; Clostridiales	OTU	13.66	0.11	0.00098	0.03136	0.32	0.18	0.55	5.72E-05	0.00011	0.53	0.31	0.9	0.01949	0.01949	2.53	28.28	NA	NA	0.27	0.08	0.91	NA	NA	1.07	0.41	2.8	NA	NA
368025	Firmicutes; Clostridia; Clostridiales	OTU	6.91	0.64	0.00016	0.01157	0.39	0.24	0.63	0.00011	0.00011	0.39	0.25	0.61	4.49E-05	8.97E-05	0.44	0.69	0.508076	1	0.6	0.18	1.99	0.40051	0.80102	1.24	0.48	3.19	0.6609	0.801
369994	Firmicutes; Clostridia; Clostridiales	OTU	1.26	0.19	4.14E-05	0.00486	0.15	0.07	0.3	1.44E-07	2.88E-07	0.36	0.19	0.71	0.00321	0.00321	0.09	1.28	0.615767	1	0.61	0.16	2.29	0.46232	0.92464	0.94	0.25	3.57	0.9298	0.9298
509416	Firmicutes; Clostridia; Clostridiales	OTU	11.46	0.01	0.00029	0.01624	0.62	0.49	0.79	7.75E-05	0.00016	0.89	0.71	1.11	0.28304	0.28304	3.84	0.29	0.49233	1	0.77	0.46	1.28	0.31148	0.62296	1.06	0.74	1.52	0.7596	0.7596
649107	Firmicutes; Clostridia; Clostridiales	OTU	1.81	0.07	0.00049	0.02147	0.5	0.34	0.73	0.0003	0.00061	0.96	0.68	1.35	0.80402	0.80402	0.41	0.72	0.002163	0.296285	0.15	0.04	0.48	0.00171	0.00342	0.32	0.13	0.79	0.0138	0.0138
214036	Firmicutes; Clostridia; Clostridiales; [Mogibacteriaceae]	Family	8.36	0.09	0.00134	0.00958	0.65	0.52	0.82	0.00022	0.00044	0.8	0.65	0.99	0.04128	0.04128	2.13	1.34	0.153306	0.475759	1.41	0.83	2.41	0.20401	0.20401	1.41	0.96	2.07	0.0777	0.1554
644244	Firmicutes; Clostridia; Clostridiales; Christensenellaceae	Family	124.55	0.05	0.00103	0.00855	0.59	0.39	0.88	0.01063	0.01063	0.51	0.34	0.74	0.0005	0.001	9.02	12.88	0.012845	0.072255	0.24	0.11	0.55	0.00075	0.00149	0.86	0.48	1.56	0.6248	0.6248
644244	Firmicutes; Clostridia; Clostridiales; Christensenellaceae	Genus	129.97	0.04	0.00337	0.03273	0.62	0.41	0.95	0.02607	0.02607	0.53	0.36	0.79	0.0016	0.0032	9.34	14.42	0.033328	0.272685	0.26	0.11	0.63	0.00287	0.00574	0.88	0.47	1.66	0.6973	0.6973
644244	Firmicutes; Clostridia; Clostridiales; Christensenellaceae	Species	132.21	0.05	0.00245	0.03479	0.61	0.4	0.93	0.02078	0.02078	0.52	0.35	0.77	0.00113	0.00225	9.64	16.05	NA	NA	0.28	0.12	0.65	NA	NA	0.91	0.49	1.7	NA	NA
234447	Firmicutes; Clostridia; Clostridiales; Christensenellaceae	OTU	0.69	1.48	5.05E-05	0.00504	0.16	0.08	0.35	3.87E-06	7.75E-06	0.29	0.14	0.61	0.00117	0.00117	0.03	0.27	0.880204	1	0.85	0.3	2.39	0.75999	1	0.82	0.24	2.84	0.7534	1
4410097	Firmicutes; Clostridia; Clostridiales; Christensenellaceae	OTU	1.99	0.1	3.37E-06	0.00086	0.13	0.06	0.28	1.46E-07	2.93E-07	0.29	0.14	0.61	0.00092	0.00092	0.26	6.45	0.751192	1	0.44	0.12	1.55	0.19955	0.3991	0.45	0.12	1.76	0.2518	0.3991
536584	Firmicutes; Clostridia; Clostridiales; Christensenellaceae	OTU	5.65	0.16	5.82E-06	0.00116	0.14	0.07	0.3	3.05E-07	6.11E-07	0.3	0.14	0.61	0.00096	0.00096	0.35	10.15	0.613865	1	0.44	0.12	1.58	0.20755	0.4151	0.49	0.13	1.92	0.3082	0.4151
555945	Firmicutes; Clostridia; Clostridiales; Clostridiaceae	Family	316.52	0.02	6.72E-08	3.36E-06	0.54	0.43	0.68	1.24E-07	2.48E-07	0.62	0.5	0.77	1.05E-05	1.05E-05	41.1	0.27	0.158586	0.475759	0.65	0.42	1.01	0.0555	0.11101	1	0.74	1.36	0.9933	0.9933
780650	Firmicutes; Clostridia; Clostridiales; Clostridiaceae	Genus	38.64	0.1	0.00185	0.02434	0.61	0.46	0.8	0.00041	0.00082	0.86	0.67	1.12	0.27672	0.27672	6.68	0.35	0.268729	0.711342	0.65	0.39	1.08	0.09858	0.19716	0.97	0.68	1.39	0.8854	0.8854
780650	Firmicutes; Clostridia; Clostridiales; Clostridiaceae	Species	37.34	0.08	0.00211	0.03382	0.61	0.46	0.8	0.00045	0.00091	0.85	0.66	1.11	0.23822	0.23822	6.62	0.31	0.190346	0.685029	0.6	0.36	1.01	0.05491	0.10983	0.91	0.63	1.3	0.5948	0.5948
780650	Firmicutes; Clostridia; Clostridiales; Clostridiaceae	OTU	13.52	0.15	0.00153	0.04055	0.31	0.16	0.58	0.00029	0.00058	0.86	0.47	1.59	0.63024	0.63024	1.54	2.37	0.042059	0.88362	0.23	0.08	0.64	0.00479	0.00958	0.68	0.32	1.45	0.3208	0.3208
199388	Firmicutes; Clostridia; Clostridiales; Clostridiaceae; Clostridium	OTU	0.28	0	0.00025	0.0148	2.67	1.66	4.3	5.66E-05	0.00011	1.85	1.15	2.97	0.01089	0.01089	0.11	0.13	0.827672	1	0.74	0.23	2.36	0.61013	1	1	0.41	2.46	0.9984	1
235424	Firmicutes; Clostridia; Clostridiales; Clostridiaceae; Clostridium	OTU	0.44	0.09	0.00106	0.03295	0.27	0.14	0.55	0.00027	0.00053	0.82	0.43	1.56	0.54432	0.54432	0.05	0.21	0.822296	1	0.7	0.19	2.62	0.59507	1	0.79	0.21	3.03	0.7312	1
555945	Firmicutes; Clostridia; Clostridiales; Clostridiaceae; SMB53	Genus	141.59	0.01	0.00022	0.00428	0.44	0.3	0.67	8.25E-05	0.00017	0.56	0.38	0.82	0.00268	0.00268	15.89	1.63	0.326943	0.779301	0.59	0.3	1.17	0.13183	0.26365	0.99	0.62	1.6	0.9745	0.9745
555945	Firmicutes; Clostridia; Clostridiales; Clostridiaceae; SMB53	Species	140.28	0.01	0.00027	0.00685	0.45	0.3	0.68	0.00013	0.00026	0.55	0.37	0.81	0.0023	0.0023	15.98	1.11	0.1998	0.685029	0.52	0.27	1.02	0.05725	0.11449	0.93	0.58	1.51	0.7831	0.7831
555945	Firmicutes; Clostridia; Clostridiales; Clostridiaceae; SMB53	OTU	130.03	0.03	0.00076	0.0286	0.45	0.29	0.69	0.00027	0.00055	0.55	0.36	0.83	0.00457	0.00457	14.84	0.91	0.215951	1	0.57	0.27	1.2	0.14198	0.28396	1.17	0.69	2	0.5624	0.5624
237991	Firmicutes; Clostridia; Clostridiales; Dehalobacteriaceae	Family	1.24	0.14	3.10E-07	7.74E-06	0.3	0.19	0.48	1.99E-07	3.98E-07	0.48	0.32	0.73	0.00051	0.00051	0.12	0.23	0.321489	0.657592	0.44	0.14	1.44	0.17498	0.34996	1	0.42	2.35	0.9986	0.9986
237991	Firmicutes; Clostridia; Clostridiales; Dehalobacteriaceae; Dehalobacterium	Genus	1.22	0.24	1.31E-05	0.00063	0.34	0.22	0.54	4.40E-06	8.80E-06	0.54	0.35	0.83	0.00449	0.00449	0.13	0.24	0.360269	0.779301	0.41	0.11	1.61	0.20313	0.40626	1.07	0.43	2.69	0.8791	0.8791
237991	Firmicutes; Clostridia; Clostridiales; Dehalobacteriaceae; Dehalobacterium	Species	1.24	0.35	9.85E-06	0.00074	0.33	0.21	0.53	2.95E-06	5.90E-06	0.53	0.35	0.82	0.00398	0.00398	0.14	0.25	0.323833	0.777198	0.44	0.14	1.45	0.17914	0.35827	1.07	0.44	2.57	0.8856	0.8856
237991	Firmicutes; Clostridia; Clostridiales; Dehalobacteriaceae; Dehalobacterium	OTU	0.78	0.94	8.28E-09	3.72E-06	0.2	0.11	0.35	1.86E-08	3.73E-08	0.31	0.18	0.52	1.18E-05	1.18E-05	0.07	0.26	0.662098	1	0.64	0.17	2.44	0.50952	1	0.96	0.27	3.38	0.9505	1
1110135	Firmicutes; Clostridia; Clostridiales; Lachnospiraceae	OTU	1.01	1.34	2.69E-05	0.00345	0.35	0.22	0.55	7.37E-06	1.47E-05	0.45	0.29	0.7	0.0004	0.0004	0.19	0.93	0.690454	1	0.67	0.19	2.41	0.53929	1	0.69	0.23	2.06	0.5111	1
174862	Firmicutes; Clostridia; Clostridiales; Lachnospiraceae	OTU	3.83	0.03	0.00073	0.0286	0.68	0.53	0.88	0.00289	0.00579	1.06	0.84	1.34	0.60152	0.60152	1.14	0.09	0.470944	1	0.8	0.44	1.44	0.46	0.48439	0.78	0.51	1.18	0.2422	0.4844
183886	Firmicutes; Clostridia; Clostridiales; Lachnospiraceae	OTU	0.37	0.02	0.00108	0.03295	2.22	1.44	3.42	0.0003	0.00059	1.44	0.94	2.22	0.0942	0.0942	0.04	0.03	0.744245	1	0.73	0.19	2.8	0.64891	1	1.01	0.29	3.52	0.9863	1
190502	Firmicutes; Clostridia; Clostridiales; Lachnospiraceae	OTU	1.25	0.02	0.00105	0.03295	2.33	1.48	3.67	0.00024	0.00049	1.81	1.17	2.81	0.00737	0.00737	0.3	0.55	0.215468	1	2.6	0.82	8.2	0.10405	0.20811	1.79	0.69	4.61	0.2294	0.2294
198362	Firmicutes; Clostridia; Clostridiales; Lachnospiraceae	OTU	0.8	0.04	0.00094	0.03109	1.88	1.35	2.62	0.00019	0.00038	1.47	1.06	2.03	0.0202	0.0202	0.12	0.19	0.649176	1	1.45	0.51	4.11	0.48929	0.97858	0.87	0.37	2.09	0.761	0.9786
310490	Firmicutes; Clostridia; Clostridiales; Lachnospiraceae	OTU	0.36	0.34	0.00039	0.01884	1.23	0.76	1.99	0.40106	0.40106	2.24	1.44	3.49	0.00035	0.00071	0.07	0.12	0.344693	1	1.57	0.43	5.76	0.49543	0.49543	2.12	0.69	6.48	0.1888	0.3776
510843	Firmicutes; Clostridia; Clostridiales; Lachnospiraceae	OTU	0.5	5.41	0.00035	0.0179	0.21	0.1	0.44	4.11E-05	8.21E-05	0.34	0.16	0.69	0.00316	0.00316	0.07	0.57	0.306761	1	1.07	0.28	4.02	0.92465	0.92465	2.2	0.58	8.38	0.2472	0.4945
517583	Firmicutes; Clostridia; Clostridiales; Lachnospiraceae	OTU	0.55	0.22	0.00042	0.01999	1.55	1.04	2.31	0.02954	0.02954	2.1	1.44	3.06	0.0001	0.00021	0.13	0.12	0.496165	1	0.73	0.22	2.43	0.60907	0.90487	1.42	0.57	3.56	0.4524	0.9049
525142	Firmicutes; Clostridia; Clostridiales; Lachnospiraceae	OTU	1.29	0.12	0.00037	0.0183	1.99	1.37	2.9	0.00034	0.00069	1.13	0.78	1.63	0.50894	0.50894	0.22	0.93	0.530331	1	1.91	0.56	6.55	0.30472	0.60945	1.2	0.42	3.41	0.7326	0.7326
529905	Firmicutes; Clostridia; Clostridiales; Lachnospiraceae	OTU	5.27	0.02	0.0003	0.01624	1.51	1.22	1.86	0.00012	0.00025	1.39	1.14	1.7	0.00116	0.00116	0.87	0.14	0.979077	1	1.07	0.57	1.98	0.83827	1	1.04	0.67	1.62	0.8661	1
591118	Firmicutes; Clostridia; Clostridiales; Lachnospiraceae	OTU	0.88	0.22	9.73E-05	0.0076	0.17	0.08	0.36	6.24E-06	1.25E-05	0.53	0.25	1.12	0.09798	0.09798	0.02	0.17	0.874144	1	1	0.36	2.79	1	1	1.23	0.36	4.24	0.7435	1
1078587	Firmicutes; Clostridia; Clostridiales; Lachnospiraceae; Blautia	Genus	1362.77	0.14	0.00174	0.02434	1.37	1.15	1.64	0.00055	0.00111	1.1	0.93	1.3	0.26605	0.26605	206.14	0.01	0.263381	0.711342	1.27	0.93	1.75	0.13546	0.27093	1.13	0.91	1.41	0.2685	0.2709
175509	Firmicutes; Clostridia; Clostridiales; Lachnospiraceae; Blautia	OTU	1.59	0.01	0.00135	0.03826	1.68	1.24	2.26	0.00075	0.00149	1.58	1.19	2.11	0.00168	0.00168	0.19	0.14	0.417949	1	0.66	0.21	2.05	0.47136	0.83387	1.39	0.63	3.1	0.4169	0.8339
184561	Firmicutes; Clostridia; Clostridiales; Lachnospiraceae; Blautia	OTU	2.12	0.32	0.00043	0.02	0.89	0.62	1.28	0.5397	0.5397	0.51	0.36	0.72	0.00015	0.0003	0.27	1.71	0.244933	1	0.77	0.28	2.12	0.61872	0.61872	1.57	0.77	3.2	0.2133	0.4266
329274	Firmicutes; Clostridia; Clostridiales; Lachnospiraceae; Blautia	OTU	0.42	0.73	0.00018	0.01182	4.09	1.98	8.44	0.00014	0.00028	1.3	0.64	2.65	0.47546	0.47546	0.17	2.79	0.750857	1	0.51	0.14	1.9	0.31786	0.63571	0.6	0.15	2.32	0.4584	0.6357
362037	Firmicutes; Clostridia; Clostridiales; Lachnospiraceae; Blautia	OTU	0.9	1.03	4.47E-06	0.001	4.14	2.21	7.77	9.52E-06	1.90E-05	1.22	0.66	2.26	0.53139	0.53139	0.37	4.82	0.746296	1	0.7	0.2	2.47	0.58131	1	0.89	0.31	2.59	0.836	1
366727	Firmicutes; Clostridia; Clostridiales; Lachnospiraceae; Blautia	OTU	0.82	0.22	0.00075	0.0286	2.38	1.48	3.84	0.00035	0.00071	2.13	1.34	3.38	0.00132	0.00132	0.13	0.54	0.824124	1	1.23	0.36	4.18	0.74062	1	1.33	0.48	3.68	0.5813	1
549635	Firmicutes; Clostridia; Clostridiales; Lachnospiraceae; Blautia	OTU	14.09	1.48	1.89E-09	1.59E-06	4.55	2.79	7.43	1.23E-09	2.47E-09	1.43	0.89	2.27	0.1376	0.1376	2.23	0.39	0.118852	1	0.96	0.33	2.78	0.9377	0.9377	2.05	0.9	4.64	0.0857	0.1714
583089	Firmicutes; Clostridia; Clostridiales; Lachnospiraceae; Blautia	OTU	42.74	0.18	0.00026	0.01516	2.3	1.36	3.89	0.0018	0.0018	2.86	1.73	4.72	3.89E-05	7.78E-05	6.7	2.7	0.037664	0.872045	2.25	0.78	6.45	0.13157	0.13157	2.67	1.18	6.06	0.0184	0.0369
178238	Firmicutes; Clostridia; Clostridiales; Lachnospiraceae; Coprococcus	OTU	26.86	0.01	0.0012	0.03481	0.69	0.55	0.87	0.00211	0.00422	1.02	0.82	1.28	0.84183	0.84183	4.46	0.07	0.52122	1	0.86	0.51	1.44	0.56114	0.89922	1.15	0.8	1.65	0.4496	0.8992
187569	Firmicutes; Clostridia; Clostridiales; Lachnospiraceae; Coprococcus	OTU	0.51	0.02	0.00094	0.03109	0.6	0.41	0.86	0.00601	0.01203	1.13	0.82	1.56	0.43745	0.43745	0.14	0.33	0.478134	1	0.71	0.22	2.31	0.5747	0.88026	1.41	0.59	3.38	0.4401	0.8803
842596	Firmicutes; Clostridia; Clostridiales; Lachnospiraceae; Coprococcus	OTU	8.53	0.13	0.00083	0.02984	0.52	0.37	0.72	9.24E-05	0.00018	0.71	0.52	0.97	0.03075	0.03075	1.28	0.02	0.080295	1	0.54	0.26	1.1	0.08914	0.17828	1.25	0.79	1.98	0.348	0.348
584463	Firmicutes; Clostridia; Clostridiales; Lachnospiraceae; Lachnobacterium	Genus	40.61	0.77	0.00246	0.02657	1.45	0.83	2.55	0.19149	0.19149	2.58	1.51	4.4	0.00049	0.00098	6.51	6	0.002383	0.053612	1.99	0.66	6.02	0.22337	0.22337	4.21	1.86	9.51	0.0006	0.0011
584463	Firmicutes; Clostridia; Clostridiales; Lachnospiraceae; Lachnobacterium	Species	39.96	0.82	0.00378	0.04832	1.43	0.82	2.5	0.2068	0.2068	2.48	1.46	4.2	0.00079	0.00158	6.48	6.17	0.003188	0.06375	1.87	0.67	5.22	0.22999	0.22999	3.89	1.77	8.57	0.0007	0.0015
16030	Firmicutes; Clostridia; Clostridiales; Ruminococcaceae	OTU	0.28	0.01	0.00069	0.02835	0.23	0.11	0.48	7.56E-05	0.00015	0.58	0.29	1.14	0.11366	0.11366	0.01	0.05	0.990079	1	0.96	0.35	2.69	0.94421	1	0.95	0.27	3.26	0.9301	1
174943	Firmicutes; Clostridia; Clostridiales; Ruminococcaceae	OTU	2.22	0.02	0.00157	0.041	1.32	1	1.74	0.04783	0.04783	1.62	1.25	2.09	0.00028	0.00057	0.5	0.08	0.141074	1	0.45	0.2	1.04	0.06067	0.12133	0.97	0.58	1.61	0.897	0.897
190649	Firmicutes; Clostridia; Clostridiales; Ruminococcaceae	OTU	0.74	0.34	4.33E-10	7.78E-07	0.17	0.1	0.31	1.49E-09	2.97E-09	0.3	0.18	0.5	4.75E-06	4.75E-06	0.17	0.7	0.780524	1	1.02	0.31	3.43	0.96853	1	1.29	0.49	3.41	0.6101	1
195406	Firmicutes; Clostridia; Clostridiales; Ruminococcaceae	OTU	0.62	0.01	7.57E-06	0.00136	0.17	0.09	0.34	3.70E-07	7.41E-07	0.7	0.37	1.3	0.2592	0.2592	0.08	0.64	0.433359	1	0.63	0.17	2.41	0.50051	1	1.24	0.36	4.28	0.7387	1
230479	Firmicutes; Clostridia; Clostridiales; Ruminococcaceae	OTU	0.68	0.03	0.00139	0.03826	0.24	0.12	0.48	5.52E-05	0.00011	0.46	0.24	0.9	0.02314	0.02314	0.11	0.38	0.954746	1	1.15	0.32	4.16	0.82607	1	1.04	0.34	3.17	0.9383	1
293085	Firmicutes; Clostridia; Clostridiales; Ruminococcaceae	OTU	0.36	0.25	0.00021	0.01276	0.25	0.13	0.51	9.32E-05	0.00019	0.4	0.21	0.76	0.00565	0.00565	0	0.06	0.996973	1	0.99	0.35	2.75	0.98201	1	0.98	0.28	3.36	0.9696	1

Online Supporting Material

			CDC															NYU														
Taxon ID	Taxonomy	Level	Normalized Mean	Maximum Cook's Distance	LRT pvalue	LRT qvalue	Fold Change (Obese)	Lower 95% CI (Obese)	Upper 95% CI (Obese)	pvalue (Obese)	p-Holm (Obese)	Fold Change (Over-weight)	Lower 95% CI (Over-weight)	Upper 95% CI (Over-weight)	pvalue (Over-weight)	p-Holm (Over-weight)	Normalized Mean	Maximum Cook's Distance	LRT pvalue	LRT qvalue	Fold Change (Obese)	Lower 95% CI (Obese)	Upper 95% CI (Obese)	pvalue (Obese)	p-Holm (Obese)	Fold Change (Over-weight)	Lower 95% CI (Over-weight)	Upper 95% CI (Over-weight)	pvalue (Over-weight)	p-Holm (Over-weight)
309720	Firmicutes; Clostridia; Clostridiales; Ruminococcaceae	OTU	12.85	0.13	0.00012	0.00904	0.62	0.46	0.84	0.00169	0.00169	0.55	0.42	0.73	3.50E-05	7.01E-05	2.02	0.1	0.644113	1	1.32	0.68	2.55	0.40734	0.81467	1.16	0.72	1.88	0.5425	0.8147
311499	Firmicutes; Clostridia; Clostridiales; Ruminococcaceae	OTU	0.92	2.66	4.60E-05	0.00486	2.19	1.48	3.24	8.08E-05	0.00016	1.07	0.73	1.57	0.74311	0.74311	0.08	0.22	0.980048	1	0.97	0.28	3.32	0.95626	1	0.93	0.34	2.58	0.8907	1
317135	Firmicutes; Clostridia; Clostridiales; Ruminococcaceae	OTU	0.35	0.04	0.00034	0.0179	0.53	0.33	0.84	0.00753	0.01507	1.27	0.85	1.89	0.24359	0.24359	0.03	0.09	0.925794	1	0.84	0.24	2.99	0.78706	1	0.89	0.23	3.49	0.8692	1
335670	Firmicutes; Clostridia; Clostridiales; Ruminococcaceae	OTU	0.74	0.14	1.39E-05	0.00208	0.38	0.25	0.59	8.09E-06	1.62E-05	0.53	0.36	0.78	0.00119	0.00119	0.17	0.41	0.264037	1	1.56	0.54	4.5	0.4064	0.4064	1.81	0.82	4.01	0.1417	0.2834
352014	Firmicutes; Clostridia; Clostridiales; Ruminococcaceae	OTU	1.17	0.16	6.93E-05	0.00623	0.2	0.1	0.39	3.63E-06	7.25E-06	0.4	0.21	0.77	0.00599	0.00599	0.23	1.11	0.456706	1	0.68	0.18	2.6	0.57493	0.76027	1.73	0.51	5.86	0.3801	0.7603
360761	Firmicutes; Clostridia; Clostridiales; Ruminococcaceae	OTU	12.26	0.1	2.65E-09	1.59E-06	0.1	0.05	0.19	2.34E-12	4.68E-12	0.39	0.21	0.73	0.0029	0.0029	1.46	13.52	0.274087	1	0.45	0.12	1.68	0.23684	0.47368	1.39	0.44	4.35	0.5752	0.5752
369827	Firmicutes; Clostridia; Clostridiales; Ruminococcaceae	OTU	5.93	0.26	0.00049	0.02147	0.26	0.14	0.49	3.92E-05	7.83E-05	0.51	0.28	0.95	0.03368	0.03368	0.87	6.77	0.225711	1	0.4	0.11	1.48	0.16954	0.33908	1.25	0.4	3.91	0.7014	0.7014
4356062	Firmicutes; Clostridia; Clostridiales; Ruminococcaceae	OTU	0.47	0.09	0.0018	0.045	2.11	1.27	3.49	0.00381	0.00762	1	0.6	1.64	0.98692	0.98692	0.06	0.74	1	1	1.23	0.33	4.52	0.75443	1	0.92	0.24	3.6	0.9099	1
694337	Firmicutes; Clostridia; Clostridiales; Ruminococcaceae	OTU	0.26	0.02	0.00078	0.0286	0.32	0.16	0.62	0.00087	0.00174	1.07	0.57	2	0.83213	0.83213	0.01	0.09	0.996762	1	0.99	0.35	2.76	0.9832	1	0.98	0.28	3.37	0.9733	1
720944	Firmicutes; Clostridia; Clostridiales; Ruminococcaceae	OTU	5.69	0.04	4.43E-05	0.00486	0.47	0.33	0.67	2.72E-05	5.43E-05	0.94	0.68	1.31	0.71349	0.71349	0.88	0.64	0.237152	1	0.55	0.25	1.23	0.14572	0.29143	1.13	0.66	1.93	0.6467	0.6467
191660	Firmicutes; Clostridia; Clostridiales; Ruminococcaceae; Faecalibacterium; prausnitzi	OTU	1.07	0.94	0.00115	0.03397	0.88	0.61	1.27	0.501	0.501	1.57	1.12	2.19	0.00827	0.01654	0.24	0.1	0.30837	1	0.62	0.2	1.91	0.40484	0.73476	1.45	0.65	3.26	0.3674	0.7348
520774	Firmicutes; Clostridia; Clostridiales; Ruminococcaceae; Faecalibacterium; prausnitzi	OTU	0.26	0.04	0.0014	0.03826	0.54	0.34	0.87	0.01159	0.02318	1.21	0.81	1.8	0.35738	0.35738	0.46	0.08	0.920929	1	0.88	0.42	1.81	0.72014	1	1.02	0.62	1.68	0.9229	1
297111	Firmicutes; Clostridia; Clostridiales; Ruminococcaceae; Oscillospira	OTU	2.69	0	1.10E-08	3.96E-06	0.29	0.18	0.45	7.29E-08	1.46E-07	0.31	0.2	0.48	1.10E-07	1.46E-07	0.36	0.77	0.198028	1	0.38	0.12	1.24	0.10901	0.21803	1	0.4	2.49	0.9979	0.9979
346686	Firmicutes; Clostridia; Clostridiales; Ruminococcaceae; Oscillospira	OTU	1.12	0.4	3.05E-06	0.00086	0.19	0.09	0.38	2.59E-06	3.30E-06	0.19	0.1	0.38	1.65E-06	3.30E-06	0.06	0.23	0.786041	1	0.72	0.19	2.76	0.6358	1	1	0.27	3.72	0.9955	1
2979308	Firmicutes; Clostridia; Clostridiales; Ruminococcaceae; Ruminococcus	OTU	15.23	0.23	0.00114	0.03397	1.89	1.35	2.64	0.00019	0.00038	1.46	1.06	2.01	0.01888	0.01888	1.45	6.49	0.066925	0.978082	1.68	0.76	3.75	0.20258	0.20258	2.02	1.12	3.66	0.0198	0.0395
771824	Firmicutes; Clostridia; Clostridiales; Ruminococcaceae; Ruminococcus	OTU	0.86	1.3	0.00068	0.02835	1.23	0.58	2.62	0.5923	0.5923	3.84	1.85	7.98	0.00032	0.00063	0.12	3.25	0.875658	1	0.57	0.16	2.04	0.38483	0.76965	0.62	0.16	2.43	0.4942	0.7697
42372	Firmicutes; Erysipelotrichi; Erysipelotrichales; Erysipelotrichaceae; [Eubacterium]; dolichun	Species	13.9	0.44	2.83E-05	0.00121	2.41	1.63	3.59	1.26E-05	2.51E-05	1.26	0.87	1.84	0.22202	0.22202	2.16	0.38	0.569851	0.966064	1	0.44	2.24	0.99351	0.99351	0.72	0.4	1.32	0.2953	0.5907
42372	Firmicutes; Erysipelotrichi; Erysipelotrichales; Erysipelotrichaceae; [Eubacterium]; dolichun	OTU	7.67	0.32	0.00145	0.03883	2.83	1.57	5.11	0.00055	0.00109	1.39	0.79	2.45	0.25148	0.25148	0.99	4.44	0.882067	1	0.83	0.23	2.96	0.77342	1	1	0.34	2.96	0.998	1
1111294	Proteobacteria	Phylum	549.98	0.18	4.77E-05	0.00048	1.73	1.33	2.24	3.66E-05	7.32E-05	1.09	0.85	1.39	0.51564	0.51564	85.11	0.18	0.08202	0.656163	1.42	0.96	2.1	0.07706	0.15412	0.92	0.7	1.21	0.5481	0.5481
841907	Proteobacteria; Deltaproteobacteria; Desulfovibrionales; Desulfovibrionaceae; Bilophila	OTU	1.08	0.55	7.79E-05	0.00666	5.17	2.66	10.07	1.34E-06	2.67E-06	2.82	1.48	5.37	0.00166	0.00166	0.36	1.31	0.169621	1	0.44	0.13	1.49	0.18677	0.37353	1.46	0.56	3.81	0.436	0.436
1111294	Proteobacteria; Gammaproteobacteria	Class	225.41	1.18	1.67E-07	2.91E-06	3.72	2.28	6.08	1.49E-07	2.98E-07	1.25	0.78	1.98	0.35225	0.35225	25.62	2.22	0.002047	0.017401	2.93	1.23	6.95	0.01473	0.02946	0.63	0.34	1.17	0.1457	0.1457
1111294	Proteobacteria; Gammaproteobacteria; Enterobacteriales	Order	193.05	0.98	3.01E-06	3.61E-05	4.55	2.54	8.16	3.48E-07	6.95E-07	1.66	0.95	2.88	0.07343	0.07343	18.83	3.15	0.000218	0.001674	2.19	0.79	6.06	0.12963	0.12963	0.28	0.13	0.6	0.001	0.002
1111294	Proteobacteria; Gammaproteobacteria; Enterobacteriales; Enterobacteriaceae	Family	216.72	2.49	3.29E-05	0.00055	3.67	2	6.72	2.67E-05	5.33E-05	1.18	0.67	2.1	0.56633	0.56633	16.94	2.45	0.000358	0.004029	2.36	0.9	6.24	0.08229	0.08229	0.35	0.17	0.74	0.0059	0.0117
1111294	Proteobacteria; Gammaproteobacteria; Enterobacteriales; Enterobacteriaceae	Genus	158.43	4.48	2.37E-05	0.00077	2.98	1.61	5.52	0.00051	0.00101	0.81	0.45	1.46	0.48167	0.48167	11.76	5.6	0.032626	0.272685	2.06	0.68	6.24	0.2004	0.24361	0.52	0.23	1.19	0.1218	0.2436
1111294	Proteobacteria; Gammaproteobacteria; Enterobacteriales; Enterobacteriaceae	Species	145.56	7.17	0.00013	0.00419	2.83	1.55	5.19	0.00075	0.0015	0.85	0.48	1.52	0.59274	0.59274	10.88	7.44	0.027949	0.299882	1.86	0.67	5.12	0.23095	0.23095	0.51	0.23	1.11	0.0908	0.1815
1111294	Proteobacteria; Gammaproteobacteria; Enterobacteriales; Enterobacteriaceae	OTU	139.99	3.77	0.0002	0.01265	2.74	1.53	4.93	0.00074	0.00149	0.92	0.53	1.61	0.77414	0.77414	13.29	13.51	0.005185	0.46176	2.68	0.92	7.75	0.06984	0.13969	0.54	0.24	1.25	0.151	0.151
865469	Proteobacteria; Gammaproteobacteria; Pasteurellales	Order	11.55	1.94	0.00199	0.01195	0.4	0.23	0.69	0.00094	0.00189	1.02	0.61	1.7	0.9443	0.9443	2.33	3.28	0.221749	0.71756	0.44	0.15	1.31	0.13928	0.27857	0.62	0.28	1.38	0.2388	0.2786

Supplemental Table 8. OTUs that were differentially abundant (p<0.05) between obese and healthy-weight participants in both the CDC and NYU studies. Models were adjusted for age, sex, and polyp status.

		CDC						NYU					
Taxon ID	Taxonomy	Normalized Mean	Fold Change	Lower 95% CI	Upper 95% CI	Maximum Cook's Distance	pvalue	Normalized Mean	Fold Change	Lower 95% CI	Upper 95% CI	Maximum Cook's Distance	pvalue
276149	Bacteroidetes; Bacteroidia; Bacteroidales; Porphyromonadaceae; Parabacteroides; N/	15.84	0.28	0.15	0.54	0.08	0.000125	3.88	0.31	0.1	0.93	0.44	0.037259
1074210	Firmicutes; Bacilli; Gemellales; Gemellaceae; NA; NA	0.28	1.8	1.09	2.99	0.04	0.022134	0.12	3.87	1.07	14.02	2.03	0.039341
579608	Firmicutes; Bacilli; Lactobacillales; Streptococcaceae; Streptococcus; NA	144.5	2.24	1.6	3.14	0.02	2.96E-06	27.87	3.76	1.72	8.21	11.05	0.000882
780650	Firmicutes; Clostridia; Clostridiales; Clostridiaceae; NA; NA	13.52	0.31	0.16	0.58	0.13	0.000288	1.54	0.23	0.08	0.64	2.62	0.004789
525378	Firmicutes; Clostridia; Clostridiales; Lachnospiraceae; Blautia; NA	7	1.37	1.11	1.68	0.03	0.002674	1.67	1.73	1	2.99	1.01	0.048374
305318	Firmicutes; Clostridia; Clostridiales; Lachnospiraceae; NA; NA	6.68	0.53	0.37	0.77	0.02	0.000799	1.02	0.15	0.06	0.39	1.54	7.61E-05
4369434	Firmicutes; Clostridia; Clostridiales; Lachnospiraceae; NA; NA	1.62	1.51	1.1	2.09	0.06	0.011925	0.29	0.26	0.09	0.79	0.09	0.017779
584137	Firmicutes; Clostridia; Clostridiales; Lachnospiraceae; NA; NA	133.29	0.7	0.5	0.99	0.06	0.042424	18.26	0.35	0.17	0.7	1.74	0.002962
592170	Firmicutes; Clostridia; Clostridiales; Lachnospiraceae; NA; NA	0.9	1.41	1	1.99	0.04	0.049653	0.15	0.29	0.09	0.94	0.17	0.038908
308057	Firmicutes; Clostridia; Clostridiales; NA; NA; NA	0.74	0.65	0.46	0.92	0.02	0.016024	0.18	0.27	0.09	0.83	0.09	0.022362
352902	Firmicutes; Clostridia; Clostridiales; NA; NA; NA	2.41	0.44	0.23	0.83	0	0.010597	1.05	0.26	0.08	0.88	6.96	0.030464
365621	Firmicutes; Clostridia; Clostridiales; NA; NA; NA	1.72	0.67	0.49	0.91	0.04	0.01081	0.39	0.29	0.11	0.75	0.25	0.011212
369822	Firmicutes; Clostridia; Clostridiales; NA; NA; NA	4.5	0.63	0.46	0.87	0.01	0.004543	0.77	0.18	0.08	0.43	0.03	0.00011
566434	Firmicutes; Clostridia; Clostridiales; NA; NA; NA	4.58	0.39	0.22	0.69	0.04	0.001048	0.46	0.22	0.06	0.84	5.13	0.027055
649107	Firmicutes; Clostridia; Clostridiales; NA; NA; NA	1.81	0.5	0.34	0.73	0.07	0.000303	0.41	0.15	0.04	0.48	0.85	0.001709
194654	Firmicutes; Clostridia; Clostridiales; Ruminococcaceae; NA; NA	2.49	0.61	0.45	0.82	0.03	0.001017	0.45	0.35	0.14	0.85	0.24	0.020072
332185	Firmicutes; Clostridia; Clostridiales; Ruminococcaceae; NA; NA	9.83	0.68	0.48	0.98	0.04	0.037127	1.08	0.33	0.14	0.77	0.63	0.010429
352347	Firmicutes; Clostridia; Clostridiales; Ruminococcaceae; NA; NA	5.21	0.76	0.59	0.97	0.02	0.025141	1.17	0.5	0.26	0.96	0.12	0.038386
188316	Firmicutes; Clostridia; Clostridiales; Ruminococcaceae; Oscillospira; NA	7.39	0.64	0.45	0.9	0.03	0.009455	1.06	0.28	0.13	0.63	0.06	0.001824
